# Viscosity of Ionic Liquids: Application of the Eyring’s Theory and a Committee Machine Intelligent System

**DOI:** 10.3390/molecules26010156

**Published:** 2020-12-31

**Authors:** Seyed Pezhman Mousavi, Saeid Atashrouz, Menad Nait Amar, Abdolhossein Hemmati-Sarapardeh, Ahmad Mohaddespour, Amir Mosavi

**Affiliations:** 1 Department of Petroleum Engineering, Shahid Bahonar University of Kerman, Kerman 76169-13439, Iran; mousavipezhman_2013@yahoo.com; 2 Department of Chemical Engineering, Amirkabir University of Technology (Tehran Polytechnic), Hafez 424, Tehran 15875-4413, Iran; s.atashrouz@gmail.com; 3 Département Etudes Thermodynamiques, Division Laboratoires, Sonatrach, Boumerdes 35000, Algeria; menad1753@gmail.com; 4 College of Construction Engineering, Jilin University, Changchun 130600, China; 5 College of Engineering and Technology, American University of the Middle East, Dasman, Kuwait; 6 Faculty of Civil Engineering, Technische Universität Dresden, 01069 Dresden, Germany; 7 School of Economics and Business, Norwegian University of Life Sciences, 1430 Ås, Norway; 8 John von Neumann Faculty of Informatics, Obuda University, 1034 Budapest, Hungary

**Keywords:** ionic liquids, viscosity, Eyring’s theory, artificial neural networks, machine intelligent system, CMIS modeling, artificial intelligence, machine learning

## Abstract

Accurate determination of the physicochemical characteristics of ionic liquids (ILs), especially viscosity, at widespread operating conditions is of a vital role for various fields. In this study, the viscosity of pure ILs is modeled using three approaches: (I) a simple group contribution method based on temperature, pressure, boiling temperature, acentric factor, molecular weight, critical temperature, critical pressure, and critical volume; (II) a model based on thermodynamic properties, pressure, and temperature; and (III) a model based on chemical structure, pressure, and temperature. Furthermore, Eyring’s absolute rate theory is used to predict viscosity based on boiling temperature and temperature. To develop Model (I), a simple correlation was applied, while for Models (II) and (III), smart approaches such as multilayer perceptron networks optimized by a Levenberg–Marquardt algorithm (MLP-LMA) and Bayesian Regularization (MLP-BR), decision tree (DT), and least square support vector machine optimized by bat algorithm (BAT-LSSVM) were utilized to establish robust and accurate predictive paradigms. These approaches were implemented using a large database consisting of 2813 experimental viscosity points from 45 different ILs under an extensive range of pressure and temperature. Afterward, the four most accurate models were selected to construct a committee machine intelligent system (CMIS). Eyring’s theory’s results to predict the viscosity demonstrated that although the theory is not precise, its simplicity is still beneficial. The proposed CMIS model provides the most precise responses with an absolute average relative deviation (AARD) of less than 4% for predicting the viscosity of ILs based on Model (II) and (III). Lastly, the applicability domain of the CMIS model and the quality of experimental data were assessed through the Leverage statistical method. It is concluded that intelligent-based predictive models are powerful alternatives for time-consuming and expensive experimental processes of the ILs viscosity measurement.

## 1. Introduction

The attention in green chemical technologies has resulted in the growth of a new class of highly tunable and special compounds named ionic liquids (ILs) [1]. Ionic liquids (ILs) were first introduced in 1914 by having to report the physical characteristics of ethyl-ammonium nitrate $\left(\left[{\mathrm{NHHH}}_{2}\right]\left[{\mathrm{NO}}_{3}\right]\right)$ [2]. Basically, ILs are formed by the combination of organic cations and organic or inorganic anions [3]. The wide types of cations are pyridinium, phosphonium, imidazolium, and ammonium. Moreover, the commonly applied class of anions includes phosphates, halides, and sulfates [2,4]. These types of liquids can preserve their state at room temperature—which results in molten salts—and, thus, are very useful in a number of temperature-sensitive processes such as biocatalysts, etc. [5].

The features of ILs depend on a number of factors, such as the extent of the cation and anion parts, cation and anion types, and the number of branches in the elements [6]. One of the most critical properties of ILs is tuneability that allows them to acquire many of their desired features by a proper combination of anions and/or cations, side chains, and task-specific groups [6]. Ionic liquids also exhibit incredibly low volatility, hence reducing air pollution once implemented [7]. Some of the other individual features of ILs are thermal and chemical stability, nonflammability, high heat capacity, unique permittivity, high ion conductivity, and good electrical, high viscosity, electrochemical stability, ease of recycling, high solubility, and high solvability capacity for both polar and nonpolar compounds [4,7,8,9,10]. Some of the primary industrial utilization of ILs include applications in lithium batteries as eco-friendly electrolytes and dissolving various compounds of organometallic. Other broad usages of ILs touch different fields such as chemical synthesis, absorption, nanomaterials synthesis, catalytic reactions, enhanced oil recovery, bioremediation, biotechnology, and electrolytes in batteries, bio-enzyme/catalysis stabilization, and membrane separation technology [11,12,13,14,15,16,17,18,19].

The study of the chemical and physical characteristics of ILs is crucial due to their high sensitivity to the addition of small quantities of impurities such as halides or water [20,21]. These characteristics include density, speed of sound, surface tension, refractive index, electrical conductivity, and viscosity. To judge their purity, the exact measurements of these properties can always be applied [20,21]. Among the properties described above is viscosity as one of the most crucial physicochemical properties that help measure the purity of ILs [21,22,23] and help understand fluid dynamics or measuring intermolecular forces [24,25,26,27,28]. Acquiring high viscosity is required in applications such as lubricants and supported membrane separation processes, while low viscosity values are typically acceptable to increase mass transfer rates and minimize pumping costs [29,30].

In general, ILs show a wide range of viscosity from 10-105 CP, while the viscosity of ILs is two to three times higher than that of traditional organic compounds [31]. For instance, the viscosity of 1-hexyl-3-methylimidazolium bis[(trifluoromethyl)sulfonyl]imide $\left(\left[C_{6}\mathrm{mim}\right]\left[{\mathrm{Tf}}_{2}N\right]\right)$ is 70 CP, while the toluene viscosity at room temperature is 0.6 CP [32]. Therefore, high viscosity ILs are suitable to be used as the stationary phase of gas and liquid chromatography, lubricants, and so on. [32]. Overall, experimental measurements of thermodynamic characteristics, chemical, and physical properties of ILs, such as viscosity in an extensive range of temperature and pressure, is more important. It is also not always feasible to calculate the proprieties of ILs because they are usually costly and time-consuming [33]. Therefore, it is necessary to develop modern and reliable predictive approaches to predict the physicochemical properties of ILs [34].

Various computational methods such as group contribution methods (GCM), quantitative structure−property relationships (QSPR), and intelligent approaches (IA) can be used to predict the viscosity of ILs [35,36]. To this end, Gardas and Coutinho [37] performed a modeling investigation of viscosity of ILs by applying GCM for 500 data points from 29 ILs (based on imidazolium, pyrrolidinium, and pyridinium) in a wide range of temperature (293–393 K). The result denotes that the absolute average relative deviation (AARD) for predicting the viscosity of ILs is equal to 7.7%. Gharagheizi et al. [38] performed another study to estimate the ILs viscosity by creating a group contribution model for 443 different ILs (1672 data points) at atmospheric and a wide range of temperature from 253.15 to 433.15 K where they obtained AARD of about 6.32%. Lazzús et al. have obtained a linear model to forecast the ILs viscosity based on GCM at the temperature range of 253–395 K where AARD of the model obtained was about 4.5% [39]. A group contribution method (GCM) based on feed-forward neural network (FF-NN) to estimate the viscosity of ILs has been proposed by Paduszynski et al. [40] for 13,000 data points (1484 ILs) where pressure and temperature ranged from 0.06–350 MPa and 253–573 K, respectively, and AARD was about 11.4%. Zhao et al. [41] proposed nonlinear (support vector machine) and linear (multiple linear regression) QSPR models to model 1502 experimental data points (89 ILs) in a wide range of temperature (253.15–395.2 K) and pressure (0.1–300 MPa), where AARD for linear and nonlinear models were obtained 10.68% and 6.58%, respectively. Therefore, proposing new, accurate models to predict the viscosity of ILs yet to be accomplished.

In this paper, 2813 experimentally obtained viscosity values for 45 ILs were gathered to establish three kinds of predictive models. The first approach includes obtaining a simple correlation (Model (I)) based on temperature, pressure, molecular weight, critical volume, boiling temperature, critical temperature, critical pressure, and acentric factor. On the other hand, five advanced models with higher accuracies were developed to forecast the viscosity at different pressures and temperatures based on Model (II) (thermodynamic properties) and Model (III) (chemical structure) by using intelligent models comprising multilayer perceptron networks optimized by Levenberg–Marquardt algorithm (MLP-LMA) and Bayesian Regularization (MLP-BR), decision tree (DT), least-square support vector machine optimized by bat algorithm (BAT-LSSVM), and committee machine intelligent system (CMIS). Furthermore, Eyring’s theory (ET) was used for estimating the viscosity of ILs containing pure systems based on temperature and boiling temperature. It should be noted that various graphical and statistical criteria were considered to investigate the reliability of the proposed approaches in order to obtain the most accurate approach. 

## 2. Viscosity Data of Ionic Liquids

**Figure 1:**
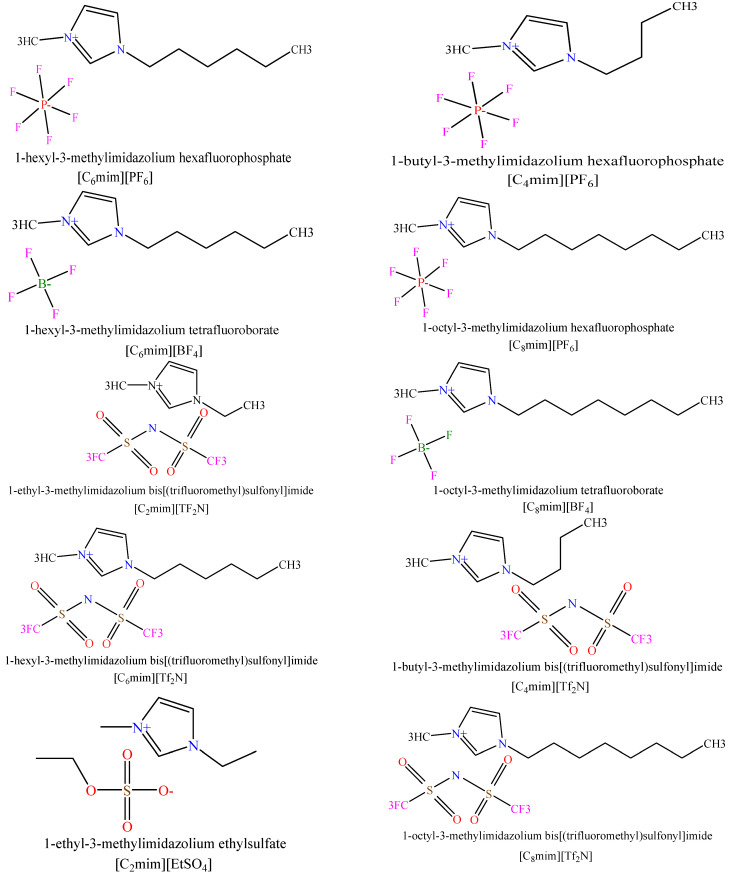

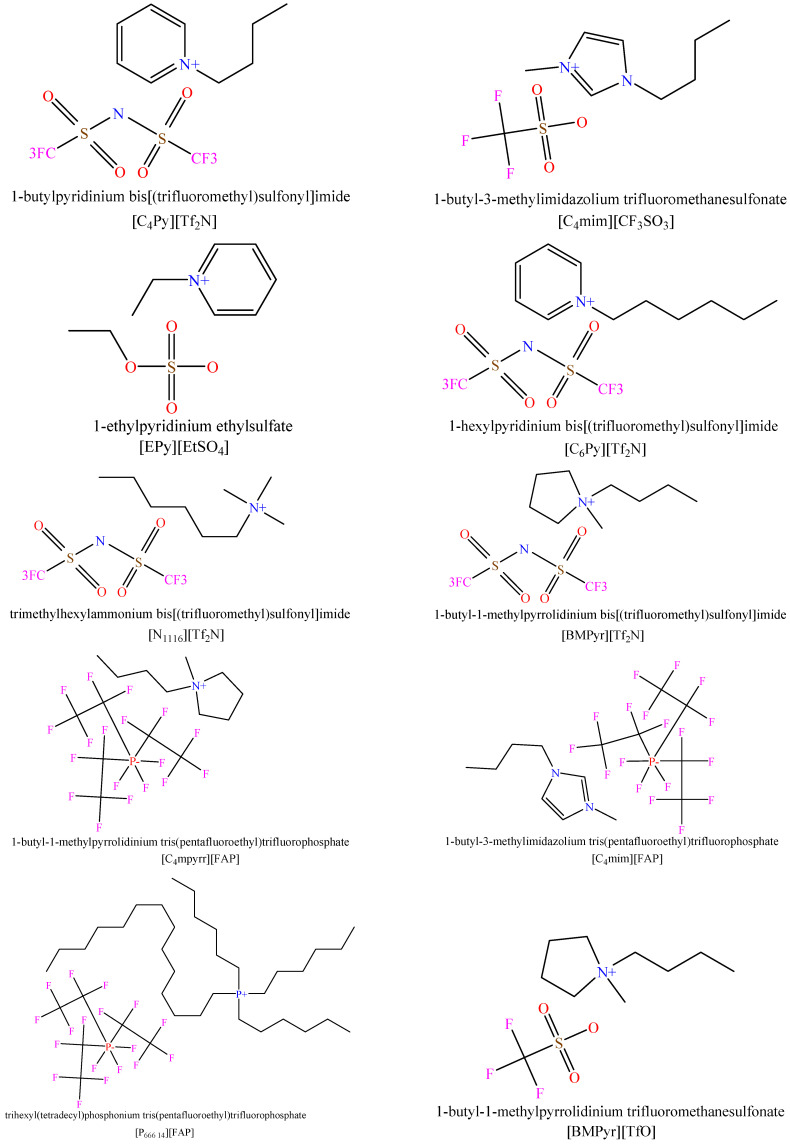

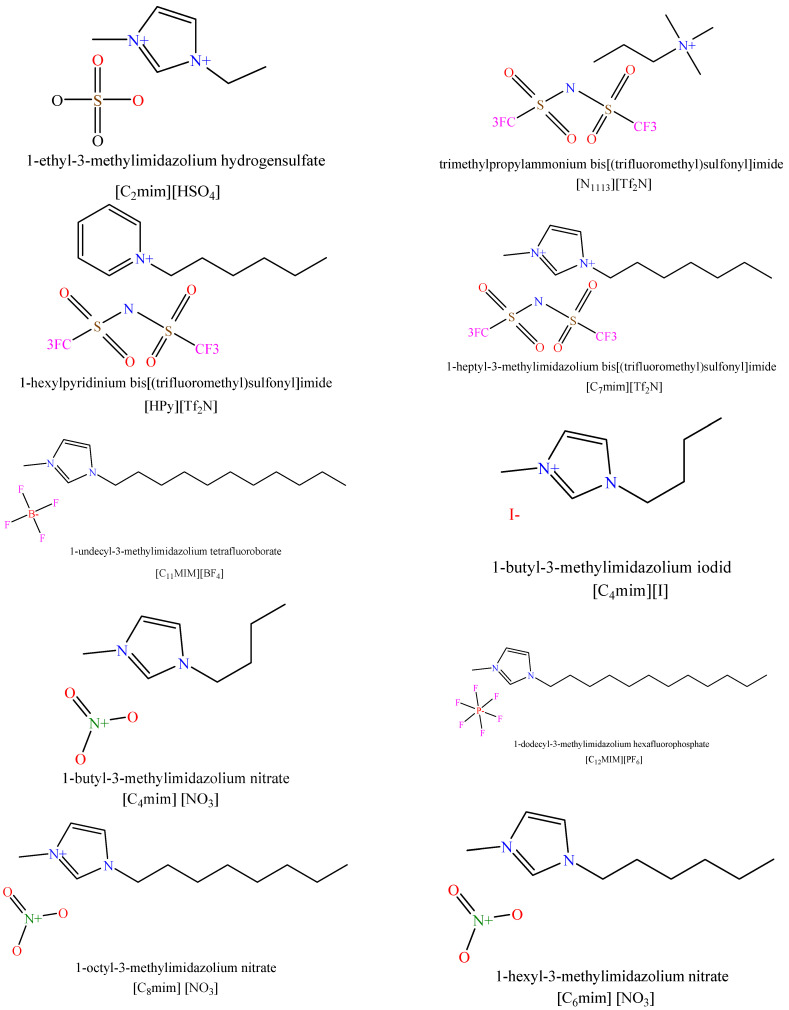
Chemical structure of ionic liquids (ILs) in this study.

The generalization and accuracy of a model highly depend on the variety and the number of data points involved in its development. For this aim, a databank—including 2813 experimental viscosity data from 45 ILs of different bases in a broad range of pressures, 0.06–298.9 (MPa), temperatures, 253.15–573 (K), and viscosities of pure ILs, 1.13–9667.6 (MPa.s)—was used to build the models [11,42,43,44,45,46,47,48,49,50,51,52,53,54,55,56,57,58,59,60,61]. The ILs cations consist of pyrrolidinium ${\left[\mathrm{Pyr}\right]}^{+}$, imidazolium [Im]+, ammonium ${\left[\mathrm{Am}\right]}^{+}$, phosphonium ${\left[\mathrm{Ph}\right]}^{+}$, and pyridinium ${\left[\mathrm{Py}\right]}^{+}$. Moreover, anions include hexafluorophosphate ${\left[{\mathrm{PF}}_{6}\right]}^{-}$, tetrafluoroborate ${\left[{\mathrm{BF}}_{4}\right]}^{-}$, bis[(trifluoromethyl) sulfonyl]imide ${\left[{\mathrm{Tf}}_{2}N\right]}^{-}$, ethyl sulfate ${\left[{\mathrm{EtSO}}_{2}\right]}^{-}$, trifluoromethanesulfonate ${\left[{\mathrm{CF}}_{3\;}{\mathrm{SO}}_{3}\right]}^{-}$, tris(pentafluoroethyl)trifluorophosphate ${\left[\mathrm{FAP}\right]}^{-}$, hydrogensulfate ${\left[{\mathrm{HSO}}_{4}\right]}^{-}$, trifluoromethanesulfonate ${\left[\mathrm{TFO}\right]}^{-}$, iodid ${\left[I\right]}^{-}$, nitrate ${\left[{\mathrm{NO}}_{3}\right]}^{-}$, diethylphosphate ${\left[\mathrm{DEP}\right]}^{-}$, dimethylphosphate ${\left[{\mathrm{DPO}}_{4}\right]}^{-}$, and methanesulfonate ${\left[\mathrm{Mesy}\right]}^{-}$. The chemical structures of designated ILs are presented in Figure 1. The ionic liquids (cations and anions), pressure, temperature, and abbreviations of ILs are provided in Table 1. In addition, the full characteristics (name, unit, min, max, and mean) of the databank used for modeling in this study are shown in Table 2. In this study, the viscosity is predicted by three approaches: Model I) a simple correlation model, Model II) intelligent models based on thermodynamic properties, pressure, and temperature (Equation (1)), and Model III) intelligent models based on chemical structure, pressure, and temperature (Equation (2)).

(1) $$\eta =f\left(T,\;P,M_{w},\;T_{c},T_{b},\;P_{c},\;\omega ,\;V_{c}\right)$$

*η* = *f* (*T*, *P*, Chemical structure) (2)

## 3. Model Development

### 3.1. Calculation of Pure Viscosity Based on Eyring’s Theory-ET

**Figure 2:**
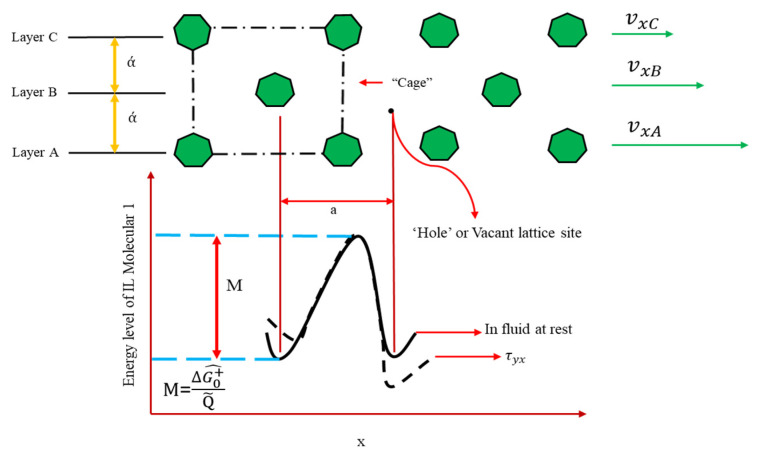
Illustration of an escape process in the flow of a liquid. Molecule 1 must pass through a “bottleneck” to reach the vacant site.

Kirkwood and co-workers [62] have established a robust kinetic theory about monatomic liquids’ transport characteristics. However, this theory does not lead to quick and easy results to apply. Henry Eyring and co-workers proposed the absolute rate theory [63,64,65]. The individual molecules are in constant motion in a pure liquid at rest. However, the motion is largely confined to vibration of each molecule formed by its nearest neighbors because of the close packing inside a “cage”. This “cage” is demonstrated by an energy barrier of height $\frac{\Delta \hat{G_{0}^{+}}}{N_{A}}$. Where $N_{A}$ stands for the Avogadro number (molecules/g-mol). To “escape” from the cage in the stationary fluid, a molar free energy of activation is needed that is denoted by $\Delta \hat{G_{0}^{+}}$ here (see Figure 2). 

Based on Eyring’s theory (ET), a molecule escapes from its “cage” into an attached “hole” in a resting liquid. Thus, the molecules move in each of the direction in jumps of length “ά” at a frequency “f” per molecule. The rate expression determines the frequency:

(3) $$f=\frac{KT}{p}\;\exp\left(-\frac{\Delta \hat{G_{0}^{+}}}{TR}\right)$$

where *K* denotes the Boltzmann constant (J/K), *P* is the Planck constant (J·s), while *R* is the gas constant (J/mole·k). *T* and $\Delta \hat{G_{0}^{+}}$ are the absolute temperature (K) and the molar activation energy in the fluid at rest, respectively. The frequency of molecular reconfigurations is increased in a fluid flowing in the x-direction with a gradient of velocity ($\frac{dv_{x}}{dy}$). Figure 2 shows the potential energy barrier as distorted under the applied stress $\tau_{yx}$ that represents as following equation:

(4) $$-\Delta \hat{G^{+}}=\Delta \hat{G_{0}^{+}}\pm \left(\gamma /ά\right)\left(\frac{\tau_{yx}\;\tilde{Q}}{2}\right)$$

in which $\pm \left(\gamma /ά\right)\left(\frac{\tau_{yx}\;\tilde{Q}}{2}\right)$ shows the estimation of the work done on the molecules. $\tilde{Q}$ denotes the volume of a mole of liquid. The frequency of forward jumps and backward jumps are “$f_{+}$” and “$f_{-}$”, respectively. Then, the combination of Equations (3) and (4), is given as follows:

(5) $$f=\frac{KT}{p}\exp\left(-\frac{\Delta \hat{G_{0}^{+}}}{TR}\;\right)\exp\left(\frac{\pm \gamma \tau_{yx}\;\tilde{Q}}{2ά\;TR}\right)$$

The net velocity that determines how far the molecules in layer “A” goes of those in layer “B” (Figure 2) is the distance traveled in each jump (ά) times the net frequency of advancing jumps ($f_{+}-f_{-}$). The following equation is applied:

(6) $$f_{xA}-f_{xB}=(f_{+}-f_{-})\;\omega$$

A linear velocity profile can be seen over a very short distance “ά” between the two layers, so that:

(7) $$-\frac{dv_{x}}{dy}=\left(\gamma /ά\right)\left(-f_{-}+f_{+}\right)$$

Finally, Equations (5) and (7) are combined as follows:

(8) $$\begin{array}{c} -\frac{dv_{x}}{dy}=\left(\gamma /ά\right)\left(\frac{KT}{p}\exp\left(-\frac{\Delta \hat{G_{0}^{+}}}{TR}\;\right)\right)(\exp\left(\frac{+\gamma \tau_{yx}\;\tilde{Q}}{2ά\;TR}\right)-\exp\left(\frac{-\gamma \tau_{yx}\;\tilde{Q}}{2ά\;TR}\right)\\ \;==\left(\frac{\gamma }{ά}\right)\left(\frac{KT}{p}\exp\left(-\frac{\Delta \hat{G_{0}^{+}}}{TR}\;\right)\right)\left(2\sinh\;\frac{\gamma \tau_{yx}\;\tilde{Q}}{2ά\;TR}\right) \end{array}$$

Additionally, if $\frac{\gamma \tau_{yx}\;\tilde{Q}}{2ά\;TR}\ll 1$, then the Taylor series can be used. Eventually, the viscosity can be obtained using the following equation:

(9) $$\eta ={\left(\frac{\gamma }{ά}\right)}^{2}\;N_{A}h/\bar{Q}\exp\left(\frac{\Delta \hat{G_{0}^{+}}}{TR}\;\right)$$

The unity factor, ($\frac{ά}{\gamma }$), is a simplification that involves no loss of accuracy, as $\Delta \hat{G_{0}^{+}}$ is obtained empirically to make sure that the equation provides consistent values with experimental values. On the other hand, the calculated $\Delta \hat{G_{0}^{+}}$ (free energies of activation) through fitting Equation (9) to experimental viscosity data versus temperature are found to be nearly constant for a specific fluid and are corresponded to the internal energy of vaporization ($\Delta {\hat{U}}_{vap}=\Delta H$_vap_-RTΔZ_vap_) at the normal boiling point, as follows [63]:

(10) $$\Delta \hat{G_{0}^{+}}\approx 0.408\;\Delta {\hat{U}}_{vap}$$

By using this empiricism and setting $\frac{ά}{\gamma }$ = 1, Equation (9) becomes:

(11) $$\eta =N_{A}p/\bar{Q}\exp\left(\frac{0.408\Delta {\hat{U}}_{vap}\;}{TR}\right)$$

The Trouton’s rule provides an accurate estimation of the energy of vaporization at the normal boiling point, as follows:

(12) $$\Delta {\hat{U}}_{vap}\approx \Delta {\hat{H}}_{vap}-T_{b}R≅9.4T_{b}R$$

According to this approximation, Equation (11) becomes:

(13) $$\eta =N_{A}p/\bar{Q}\exp\left(\frac{\lambda \;T_{b}\;}{T}\right)$$

In this work, in Equation (13), a “λ” term was added for each ILs based on GRG in Excel software. This term is not constant, while changing with each ionic liquid. Equations (11) and (13) show good consistency with the apparently successful and long-used empiricism η=Aexp(B/T). The theory shows a decrease in viscosity with temperature.

### 3.2. Generalized Reduced Gradient

The generalized reduced gradient (GRG) approach is frequently used as a solver for multivariable problems. This scheme is designed to integrate and solve linear and non-linear problems based on the concept of the reduced gradients [66]. It controls the component in such a way that when the process transitions from one stage to another, the active constraints remain to be satisfied. In fact, at a given point x, GRG provides a linear estimation for the gradient. The constraints and objective gradient are simultaneously solved, and the gradient of the objective function can be represented as constraint gradients. Then, the search area becomes smaller by moving in a practical path. Following expressions denote an objective function f(z) that is subjected to h(z) [67]:

Minimize: f(z) = z (14)

(15) $$\mathrm{Subjected}\;\mathrm{to}:\;h_{k}\left(z\right)=0$$

In the following form, GRG can be adjusted [67]:

(16) $$\frac{df}{dz_{k}}=\nabla z_{k}^{t}f-\nabla z_{i}^{t}f{\left(\frac{\partial h}{\partial z_{i}}\right)}^{-1}\;\frac{\partial h}{\partial z_{k}}$$

Basically, f(z) will be minimum under two simple conditions that are df(z) = 0 or $\frac{df\left(z\right)}{dz_{k}}$ [68,69].

### 3.3. Decision Tree-DT

The first decision tree (DT) was the model first developed by Morgan and Sonquist [70], Automatic Interaction Detection (AID) that was introduced by Morgan and Sonquist [70]. This approach is a non-parametric supervised learning method that is applicable to both classification and regression problems. The first algorithm for the tree classification was THAID which Messenger and Mandell have suggested [71]. Learning and classification are two steps in the DT approaches. During the learning phase, the algorithm generates a tree from a set of training samples that have been classified. In the following step, unclassified data are classified using the tree developed in the learning phase [72]. The decision tree (DT) is successfully applied in many different fields such as speech recognition, remote sensing, radar signal classification, expert system, character recognition, and medical diagnosis. They are relatively inexpensive in terms of computing and appropriate accuracy. The decision tree is capable of breaking down a complicated decision-making process into a collection of simple decisions, thereby simplifying the decision [73]. A flow chart-like structure for the decision tree consists of branches, internal nodes, and root nodes. The whole of the sample space was shown via the top node with no income branch that is called the root node. The nodes with one incoming branch and more outgoing edges are classified as the internal nodes or test. The leaves or terminal nodes are identified as the other nodes that indicate the final results. The decision tree has made with three parts: pruning, stopping and splitting [74]. Splitting means that the data are divided into a number of subsets based on the most significant attribute testing that is also applicable to training instances. For the variance reduction, standard deviation reduction, and classification tree, the various criteria may be handled including the Gini index, information gain, classification error, gain ratio, and towing [75].

### 3.4. Multilayer Perceptron Neural Network—MLPNN

Artificial Neural Network (ANN) is modeling technique which is inspired from the human brain network as a smart computing plan. The simplest element of ANN processing is known as neurons in which connections interrelate and are organized into various layers. Neural networks are used in many aspects, including identification, estimation of functions, recognition of patterns, clustering etc. [76]. ANN has been applied in various fields, including electronics, medical, aerospace, petroleum and chemical industries [77,78]. The most popular ANN model is the multilayer perceptron (MLP). In an MLP model, there are several layers between the input and output layers, which are called hidden layers [79]. The hidden layers are specific connections between the inputs and outputs of the models. In addition, the number of neurons should be specified through a trial and error process in the hidden layer. The number of neurons in the first and last layers is regulated with input and output parameters. The use of two hidden layers in more complex problems is more appropriate than one hidden layer, but one hidden layer in the MLP model should usually be used in simple problems [80]. The value of each hidden/output neuron is calculated through the multiply and summation of the previous neurons to the neurons’ weights, and ultimately, a bias term is added to this summing-up [79]. This result is passed through the activation functions that are defined as follows:

(17) $$\mathrm{Tangsig}=\mathrm{Tan}h:f\left(n\right)=\frac{e^{n}-e^{-n}}{e^{n}+e^{-n}}=\frac{2}{1+e^{-2n}}-1$$

(18) $$\mathrm{ArcTan}:f\left(a\right)=\tan^{-1}\left(a\right)$$

(19) $$\mathrm{Logsig}\;=\;\mathrm{Sigmoid}:f\left(y\right)=\frac{1}{1+e^{-y}}$$

(20) Linear=Pureline: f(z)=z

(21) Sinusid: f(z)=sin(z)

In hidden layers, logsig and tansig are the frequently applied activation functions, while pureline is usually considered the output layer’s activation function. To illustrate how the outcomes are gained from an MLP model, consider an illustrative model with two hidden layers having logsig and tansig as transfer functions, respectively, and an output layer with pureline as the transfer function. The output of the models is achieved by:

(22) $$\mathrm{Output}=\mathrm{pureline}\left(w_{3}\times \left(\mathrm{logsig}\left(w_{2}\times \left(\mathrm{tansig}\left(x\right)+b_{1}\right)\right)+b_{2}\right)+b_{3}\right)$$

In the above-mentioned equation, pureline, logsig, and tansig are activation functions, respectively, $b_{1}$ denotes bias vectors of the first hidden layer and $b_{2}$, b_3_ are bias vectors of the second hidden layer and the output layer, respectively. Additionally, $w_{1}$, $w_{2}$, and $w_{3}$ are the matrix weight of the first layer, the matrix weight of the second layer, and the matrix weight of the output layer, respectively [80]. 

The optimization algorithm is one of the most key roles in the performance of the MLP model which are used for training the model. Thus, two main optimization algorithms have been used in this study, including LMA, Levenberg-Marquardt, and BR, Bayesian Regularization. Additional information on developing LMA and BR in the MLP training phase can be found elsewhere [81,82,83,84]. A scheme of the MLP network that was used in this study is represented in Figure S1 (in the Supplementary File).

### 3.5. Least Square Support Vector Machine—LSSVM

A support vector machine (SVM) is a supervised and powerful, intelligent tool applied on known input/output data for various purposes such as pattern recognition, problem classification, and regression analysis [85,86]. The least-square SVM (LSSVM) was proposed by Vandewalle and Suykens [87] as a newer version of the support vector machine. Further, the LSSVM has emerged to improve and prevail the typical shortcomings of the SVM approach and simplify its solution [85]. The LSSVM updates the optimization constraints and mathematically determines the regression error. In reality, regression error in SVM algorithms during the learning process is optimized, and it is numerically defined and resolved in LSSVM approaches [85]. The penalized function of the least square SVM (LSSVM) method is described as shown below [85,88,89,90]:

(23) $$Q_{LSSVM}=\lambda \times \overset{N}{\underset{k=1}{\sum }}e_{k}^{2}+\frac{w^{T}}{2}$$

where λ is the summation of regression errors, while *T* shows the transpose matrix. Equation (23) is subjected to the following constraint [85]:

(24) $$y_{k}=e_{k}+b+w^{T}\varphi \left(x_{k}\right),\;k=1,\;2\;\ldots \;N$$

where $e_{k}$ denotes the regression error of *N* training objects, *y* shows the output vector of the model, *b* is the intercept or the bias of linear regression. Additionally, *T* expresses the transposed matrix, and *w* represents linear regression slope. The weight function (*w*) is expressed as follows [85]:

(25) $$w=\sum_{k=1}^{N}x_{k}\times a_{k},\;\mathrm{where},\;a_{k}=2\times e_{k}\times \lambda$$

Where $a_{k}$ denotes the Lagrangian multiplier. λ corresponds to the relation of specific weights and also the weight of all regression errors, while $e_{k}$ represents regression error related to whole databank. Using least square SVM (LSSVM) method, another form of Equation (25) is represented as follows [85,87,88,89,90]:

(26) $$y=b+\overset{N}{\underset{k=1}{\sum }}x_{k}^{T}\times x\times a_{k}$$

Consequently, the Lagrange multipliers are described as the following equation [85]:

(27) $$a_{k}=\frac{\left(y_{k}-b\right)}{\left(2\lambda \right)+x\times x_{k}^{T}}$$

Using the following Kernel function, the aforementioned equation for linear regression can be rewritten as [85]:

(28) $$f\left(x\right)=b+\overset{N}{\underset{k=1}{\sum }}a_{k}\times k\left(x,x_{k}\right)$$

In which $k\left(x,x_{k}\right)$ shows the Kernel function. $k\left(x,x_{k}\right)$ denotes the dot product of $\Phi \left(x_{k}\right)$ and $\Phi {\left(x\right)}^{T}$ as follows [85]:

(29) $$K\left(x,x_{k}\right)=\Phi \left(x_{k}\right)\times \Phi {\left(x\right)}^{T}$$

In the present study, one of the most well-known Kernel functions, i.e., the radial basis Kernel function, was applied. This latter is defined as follows:

(30) $$K\left(x,x_{k}\right)=\exp\left(-\frac{{\left||x_{k}-x|\right|}^{2}}{\sigma^{2}}\right)$$

In the above equation, $\sigma^{2}$ is a regulation parameter that is to be obtained by an optimization algorithm. Briefly, there are two adjustable parameters in the LSSVM technique, namely λ and $\sigma^{2}$, which should be optimized during training step. The parameters for tuning were optimized using the bat (BAT) algorithm. This approach was named BAT-LSSVM. In this approach, $\sigma^{2}$ and λ are optimized using BAT algorithm for determining viscosity of ILs. The schematic representation of this approach for predicting viscosity of ILs is showed in Figure S2 (in the Supplementary File).

### 3.6. Committee Machine Intelligent System (CMIS)

In an extensive number of studies, various artificial intelligence modelling technique are employed, and after selecting the most accurate model, the other approaches are left out. The better choice is to utilize these models to build a more precise model. Many years ago, Nilsson presented a Committee Machine Intelligent System (CMIS) as a kind of artificial neural network. Through this process, the basic goal is to divide and conquer until resolving a problem. In addition, the outputs of each approach have been mixed and the benefits of all work with little extra calculation have been achieved. Afterward, the model can outperform the best single network [91,92]. As a matter of fact, in this technique, different predictive models are combined in order to form a more efficient and accurate model. Different methods have emerged for merging several models in a single model. The categories of committee machines can be divided into the two following types [93]:

(1) Static structure
(2) Dynamic structure

**Figure 3:**
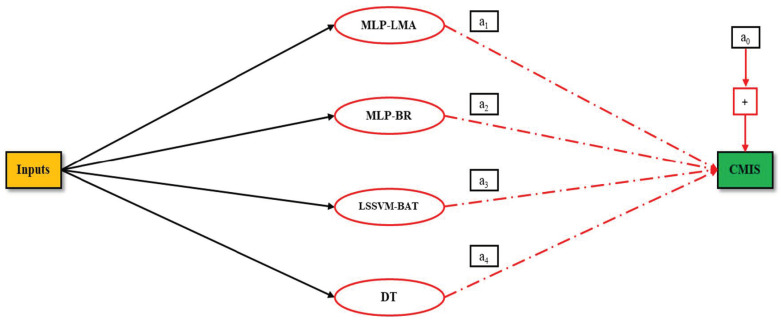
Committee Machine Intelligent System (CMIS) model offered in this study.

An appropriate approach based on simple averaging or weighted averaging is employed to linearly combine the resolutions and getting the best model [94]. Herein, an improved weighted averaging approach was used and a bias factor was added to the linear equation. Each model’s contribution extent in a CMIS corresponds to the absolute coefficient of that model in the CMIS linear equation. Figure 3 shows a scheme of the CMIS approach in this study.

### 3.7. Optimization Technique

#### Bat Algorithm (BAT)

In 2010, Yang Xinshe introduced the bat algorithm (BA) by analyzing the behaviors and the features of the microbat [95]. In the rest of this section, we shed light on the echolocation and details of this nature-inspired algorithm. Yang and Deb [96] used the idealized and approximate rules in the BA approaches with the following steps:

1. All the species of the bat utilize echolocation to sense distance, and bats ‘know’ the discrepancy among food/prey and background obstacles in some magical techniques.
2. In order to search prey, the bats can fly fortuitously with the velocity $v_{i}$ at position $x_{i}$ with a frequency $f_{\min}$, loudness $A_{0}$, and a variable wavelength λ. Bats can spontaneously adjust the wavelength and/or frequency of their generated pulses and regulate the level of pulse emission r in the range of [0,1], reliant on the nearness of their goal.
3. Although there are various methods to regulate the loudness, it is usually assumed that the loudness is between a positive $A_{0}$ and a minimum constant amount, which is represented by $A_{\min}$.

The following equations show the motion of a virtual bat by Yang’s method [97]:

(31) $$f_{i}=f_{min}-\left(f_{min}-f_{max}\right).\beta$$

(32) $$v_{i}^{t}=v_{i}^{t-1}-\left(x_{best}-x_{i}^{t}\right).f_{i}$$

(33) $$x_{i}^{t}=v_{i}^{t}+x_{i}^{t-1}$$

where *β* is between 0 and 1 and denotes a random obtained from monotonic, min and max are the suffixes that are shown minimum and maximum value, respectively. The velocity of the bat is $v_{i}$, the current iteration is indicated as “t”. The global close-best solution discovered so far over the whole population is denoted $x_{best}$. The location of the *i*th bat in the solution space is $x_{i}$ and f denotes the frequency used by the bat to seek for its hunt. Furthermore, one of the roles in the system is also assumed to be the rate of pulse emission from the bat. The symbol $r_{i}\in \left[0,1\right]$ is the pulse emission rate, also, the suffix i denotes the *i*th bat. A random number is created in each iteration and compared with $r_{i}$. The random walk is denoted a local search strategy, if $r_{i}$ is smaller than a random walk. The below-shown equation represents a new solution for the bat:

(34) $$x_{new}=rand.A^{t}+x_{old}$$

where the average loudness of all bats at the current time step is $A^{t}\langle A_{i}^{t}\rangle$, while rand ∈[−1,1]. The loudness $A_{i}$ and pulse emission rate $r_{i}$ are also updated after updating the positions of the bat, only when the close-best global solution is updated and $A_{i}$ is bigger than the random number. Equations (35) and (36) state the update of $r_{i}$ and $A_{i}$ [97]:

(35) $$A_{i}^{t+1}=A_{i}^{t}.\beta$$

(36) $$r_{i}^{t+1}=\left[-\exp\left(-\alpha t\right)+1\right]r_{i}^{0}$$

where β and α are constants. As a result, 0<β<1 and α>0 [97].

The above-described steps are reiterated until reaching a stopping condition.

## 4. Model Assessment

### 4.1. Statistical Criteria 

In order to evaluate the validity of the obtained models, the mathematical formula for statistical assessment parameters including average relative deviation (ARD%), determination coefficient ($R^{2}$), standard deviation (SD), root mean square error (RMSE), and average absolute relative deviation (AARD%) were used. These statistical parameters are detailed as follows:

#### 4.1.1. Determination Coefficient ($R^{2}$)

Regression determination is a measure of fitting quality revealing the accuracy of the model. Accordingly, if its value is close to 1, the model matches the data more accurately. The mathematical formula of $R^{2}$ is given below:

(37) $$R^{2}=\frac{\sum_{i=1}^{N_{P}}{\left(\eta_{i}^{exp}-\bar{\eta }\right)}^{2}-\sum_{i=1}^{N_{P}}{\left(\eta_{i}^{cal}-\eta_{i}^{exp}\right)}^{2}}{\sum_{i=1}^{N_{P}}{\left(\eta_{i}^{exp}-\bar{\eta }\right)}^{2}}$$

#### 4.1.2. Average Relative Deviation (ARD%)

The relative deviation of the estimated data from the experimental is measured by the percentage of ARD with the following equation:

(38) $$\mathrm{ARD}\%=\frac{100}{N_{P}}\underset{j=1}{\sum }\left(\frac{\eta_{j}^{exp}-\eta_{j}^{est}}{\eta_{j}^{exp}}\right)$$

Positive and negative ARD (%) denote underestimation and overestimation of a model, respectively.

#### 4.1.3. Standard Deviation (SD)

It is a measure of the scattering of data around the mean. It is defined as:

(39) $$\mathrm{SD}={\left(\frac{1}{N-1}\overset{N_{P}}{\underset{j=1}{\sum }}{\left(\frac{\eta_{j}^{exp}-\eta_{j}^{est}}{\lambda_{j}^{exp}}\right)}^{2}\right)}^{\frac{1}{2}}$$

#### 4.1.4. Average Absolute Relative Deviation (AARD%) 

The AARD value is a measure of the relative absolute deviation of the predicted/represented data from the actual/real data. It is represented by:

(40) $$\mathrm{AARD}\left(\%\right):100\times \frac{\sum_{j=1}^{N_{P}}\left|\frac{\eta_{j}^{exp}-\eta_{j}^{est}}{\eta_{j}^{exp}}\right|}{N_{P}}$$

#### 4.1.5. Root Mean Square Error (RMSE)

The root-mean-square error (RMSE) is a widely used calculation of the differences between values (sample or population values) expected by a model or estimator and the observed values. It is denoted by:

(41) $$\mathrm{RMSD}=\sqrt{\frac{1}{N_{P}}\overset{N_{P}}{\underset{j=1}{\sum }}{\left(\eta_{j}^{exp}-\eta_{j}^{est}\right)}^{2}}$$

where $N_{P}$ points out the numbers of data points and η is the experimental/real values of viscosity of ILs, while $\bar{\eta }$, superscripts “*est*”, and “*exp*” are the average of viscosity of ILs obtained by experiments/real data, the estimated value, and the experimental/real value, respectively.

### 4.2. Graphical Evaluation of the Models

To better assess the proposed models and analyze their predictive accuracy, several graphical plots were employed in this study. The plots include cross-plot, cumulative frequency diagram, and error distribution diagram. In the error distribution, the percent relative deviation is plotted versus the target/real values to measure the distribution of error around the zero-error line and to show whether the model has an error trend or not. Cross-plot includes sketching the estimated/represented value obtained by the model versus the experimental data. Then, between the experimental data and predicted/represented values, a straight line of a 45° (unit slope line) is drawn. Eventually, the closer the plotted data to this line, the higher is the model accuracy. For a cumulative frequency diagram, the larger part of the approximations will be in a standard error range where the cumulative frequency is calculated from the absolute relative error.

## 5. Result and Discussion

### 5.1. Development of Models

In this study, models were developed based on 2813 data points (45 ionic liquids) that were gathered from the literature. The random databank division was conducted to attain two subsets, the test (20% of the whole dataset) and training sets (80% of the whole dataset). In fact, the validity of the constructed models and over-fitting problems were monitored based on the “testing” subset (563 data points). The models’ structure and the adjustment of their tuning parameters were performed based on the “training” subset (2250 data points) [98]. 

**Figure 4:**
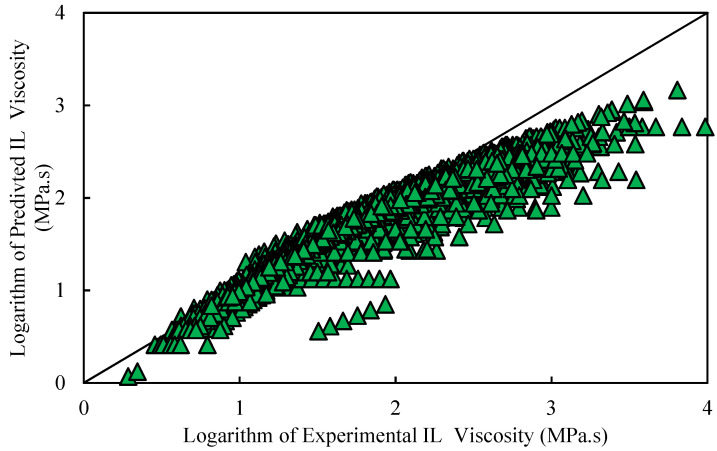
Cross plot of the proposed Eyring’s theory for viscosity of ILs.

As it was mentioned early, *T*, *P*, $M_{w}$, $V_{c}$, $T_{b}$, $T_{c}$, $P_{c}$, ω, and chemical structure were considered as the input parameters, while the output was the viscosity of ILs (MPa·s). Firstly, a new empirical correlation was established based on the GRG approach. The following relation was obtained for viscosity of ILs:

(42) $$\log\left(\eta \right)=\frac{\left(aT^{i}+bP^{j}+cM_{w}^{k}+dV_{C}^{l}+eT_{b}^{m}+fT_{c}^{n}+gP_{c}^{o}+h\omega^{p}\right)}{T^{q}}$$

where $T_{c}$ and $P_{c}$ denote the critical temperature and the critical pressure of ionic liquids, respectively. Additionally, ω, *T*, and *P* are the acentric factor, temperature, and pressure, respectively, while $M_{w}$ is the molecular weight of ILs, $V_{c}$ is the critical volume of ILs, and $T_{b}$ is the boiling temperature of the ILs. Moreover, the other parameters are the adjustable coefficients of the correlation which are shown in Table S1 (in the Supplementary File). The ARD%, AARD%, SD, RMSE, and $R^{2}$ for the proposed correlation are 13.44%, 28.34%, 0.368, 394.12, and 0.207, respectively. The cross-plots between the logarithm of predicted data were plotted against the logarithm of experimental viscosity data for the presented correlation (Equation (42)) is shown in Figure S3 (in the Supplementary File). This plot depicts a medium-uniform distribution of the predictions around the unit-slope line. On the other hand, Eyring’s theory (ET) was applied to estimate the viscosity of ILs based on boiling temperature ($T_{b}$) and temperature (*T*) for the gathered database. The achieved correlation is expressed as follows:

(43) $$\eta =N_{A}p/\bar{Q}\exp\left(\frac{\lambda \;T_{b}\;}{T}\right)$$

where η is the predicted viscosity of pure ILs (MPa·s). $N_{A}$ and *p* are the Avogadro number (${\mathrm{mole}}^{-1}$) and the Plank constant (J·s), respectively. $\bar{Q}$ denotes the volume of a mole of liquid (m^3^/mole), $T_{b}$ and *T* represent the boiling temperature (K) and temperature (K), respectively, while λ is a factor that was obtained based on GRG and for each ILs is not constant. The average absolute relative deviation (AARD) is 21.86%. Additionally, Figure 4 represents a cross-plot between the logarithm of predicted of ILs viscosity versus the logarithm of experimental of ILs viscosity. As seen, a moderate conformity was noticed as the data points were not very close to the diagonal line. The theory of Eyring is not sufficient in this study, because Arrhenius dependency does not match the experimental transport features of ILs. Viscosity values reduce significantly with temperature rises, as generally reported for all ILs measured. Furthermore, the thermally enabled transport features of ILs are generally defined by the Vogel–Tamman–Fulcher (VTF) development because of the development of an underlying complex energy landscape with a multiplicity of local potential energy minimums and a broad dispersion of energy barriers [99,100,101,102].

Further, DT, MLP and LSSVM-BAT modelling techniques were employed to predict the viscosity of ILs based on Model (II) and Model (III). In the first stage based on Model (II), the MLP training phase was performed utilizing two learning techniques, namely, BR and LMA. For the best architecture of the MLP network with BR and LMA optimizers, using three hidden layers were determined to be the most appropriate for predicting the viscosity of ILs. Therefore, each layer for MLP-LMA and MLP-BR encompasses 11-11-9 and 13-11-9 neurons, respectively. The best transfer functions for all of three layers of MLP-LMA and MLP-BR models were found as tansig. The optimum values of the main parameters of the developed LSSVM model, i.e., $\sigma^{2}$ and λ, were investigated using the BAT algorithm. For Model (II), the obtained values for $\sigma^{2}$ and λ are 25.4817 and 5,793,328.591, respectively. Table 3 reports the statistical assessment of the constructed schemes. 

According to the results presented in the Table 3, the MLP-BR model is the most reliable technique compared to the other models since it provides accurate predictions for the whole dataset. The proposed models including MLP-LMA, MLP-BR, LSSVM-BAT, and DT were combined into a CMIS. The coefficients of the constructed CMIS model were optimized through a multiple linear regression and the following equation was obtained for this latter:

(44) $$\eta_{ILs}^{i}=a+by_{DT}^{i}+cy_{LSSVM-BAT}^{i}+dy_{MLP-BR}^{i}+ey_{MLP-LMA}^{i}$$

where *a*, *b*, *c*, *d*, and *e* were determined to be 0, $8.77\times 10^{-5}$, 0.049344, 0.619175, and 0.330275, respectively.

In the other procedure based on Model (III) (chemical structure, pressure, and temperature) the MLP models with two optimization algorithms, including LMA and BR were developed. The hidden layers for MLP-LMA and MLP-BR were chosen 12-11-9 and 11-11-9, respectively, where the first, second, and third numbers correspond to the number of neurons of the first, second, and third hidden layer, respectively. Additionally, tansig was found to be the best activation function for the three hidden layers of MLP-LMA and MLP-BR models. The tuning parameters (namely λ and $\sigma^{2}$ ) are the most important in the LSSVM-BAT model. The parameters of the LSSVM method were determined as 6,635,314.0686 and 27.3672 for λ and $\sigma^{2}$, respectively, using BAT optimization algorithm. Table 4 shows the statistical parameters for predicting the viscosity of ILs based on Model (III). Accordingly, MLP with BR optimization is a better choice for viscosity estimation based on Model (III). Therefore, a CMIS method that combines the proposed paradigms, i.e., MLP-LMA, MLP-BR, LSSVM -BAT, and DT, was implemented. The appropriate coefficients of the CMIS model were specified employing a multiple linear regression. For this goal, the following correlation is provided:

(45) $$\eta_{ILs}^{i}=q+my_{DT}^{i}+ny_{LSSVM-BAT}^{i}+dy_{MLP-BR}^{i}+ey_{MLP-LMA}^{i}$$

where the coefficients q to e are as shown below:

*q* = 0.052; *m* = 0.0001; *n* = 0.151; *d* = 0.466; *e* = 0.379.

### 5.2. Statistical Evaluation

In order to indicate the error margin, various statistical indexes, including ARD, AARD, RMSE, SD, and $R^{2}$ were computed for each of the developed model. As reported in Table 3, the computed AARD, ARD, SD, and RMSE for the CMIS are small values for both test and training sets, as well as the whole dataset. The value of AARD for CMIS model based on Model (II) is 3.24%, which shows the higher accuracy of this model compared to the four ANN-based models. Briefly, Table 3 depicted that the models are ranked as follows in terms of accuracy:

CMIS > MLP-BR > MLP-LM > LSSVM-BAT > DT

In the other hand, based on Model (III), the same statistical parameters comprising ARD, AARD, RMSE, SD, and $R^{2}$ are also presented for MLP-BR, MLP-LMA, LSSVM-BAT, DT, and CMIS which are summarized in Table 4. The models are categorized according to their precision as follows: DT < LSSVM-BAT < MLP-LMA < MLP-BR < CMIS. As can be deduced, the CMIS model could provide more accurate predictions compared to the other established models.

### 5.3. Graphical Error Analysis

**Figure 5:**
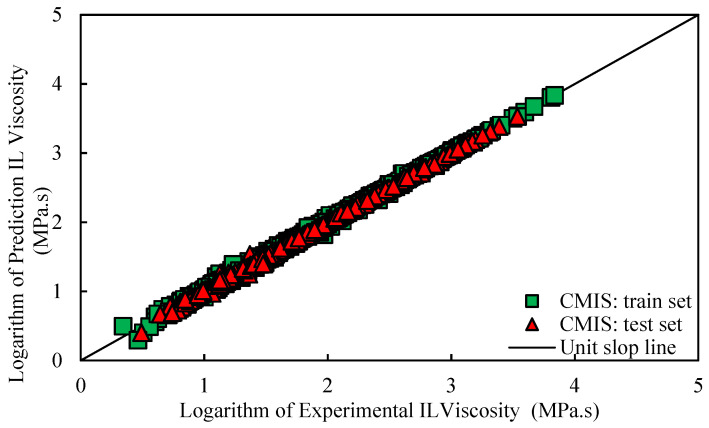
Logarithm of experimental viscosity data against predicted value based on Model (II) for CMIS model in this study.

**Figure 6:**
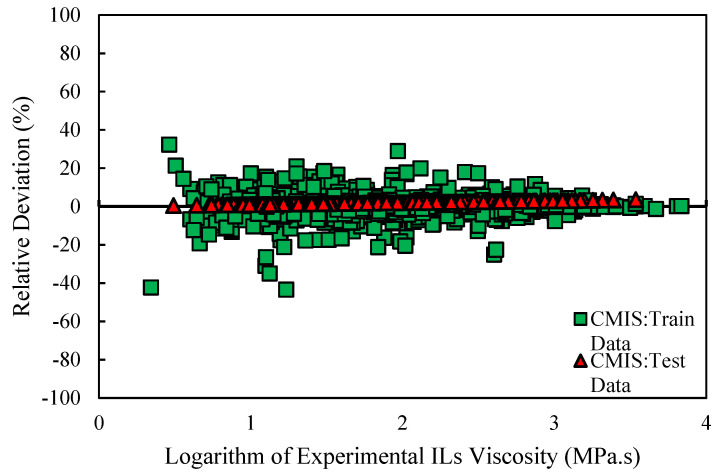
Relative deviation of prediction of CMIS model versus logarithm of experimental data based on Model (II).

Various graphical error evaluations based on Models (II) and (III) were investigated to give a more transparent view of the performance of the models. To assess the accuracy of Model (II), the logarithm of predicted viscosity data was plotted against the logarithm of corresponding experimental values in Figure 5. Therefore, Figure 5 shows comparative cross-plots for test and training sets for CMIS model (cross-plot for other models such as: MLP-BR, MLP-LMA, DT, and LSSVM-BAT are presented in Figure S4 in the supplementary File). A high-dense accumulation of the points can be seen around the unit slop for both test and training datasets. On the other hand, higher accuracy of the constructed CMIS compared to the other models for approach (II) is clearly shown in these figures. Accordingly, the CMIS paradigm is introduced as the best predictive method. Figure 6 represents the relative deviation (RD) against the experimental viscosity of ILs for the CMIS model. Additionally, Figure S5 (in the Supplementary File) reveals the experimental viscosity of ILs against the relative deviation for MLP-BR, MLP-LMA, LSSVM-BAT, and DT. As shown in these figures, for LSSVM-BAT and DT, the data distribution near the zero line is larger than the developed CMIS model. This observation confirms the results reported in Table 3. The implementation of the CMIS results in the minimum total relative deviation guiding to the narrowest error margin. 

**Figure 7:**
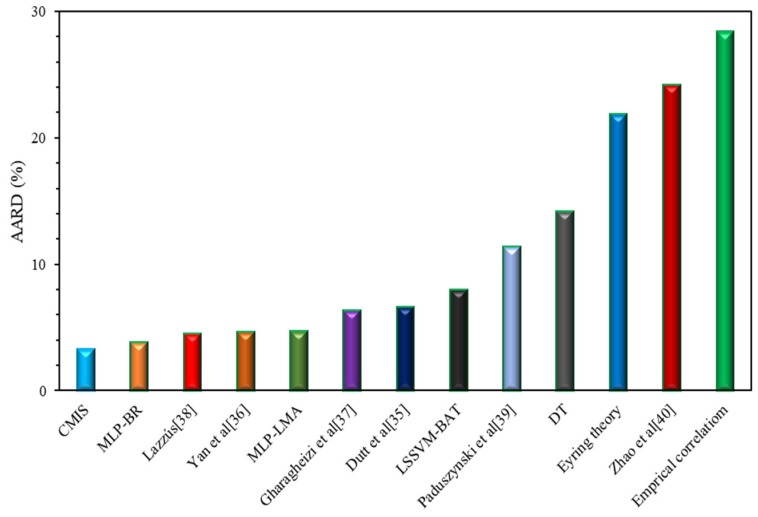
Comparison between absolute average relative deviation (AARD, %) of different models for estimation of viscosity of ILs based on Model (II).

**Figure 8:**
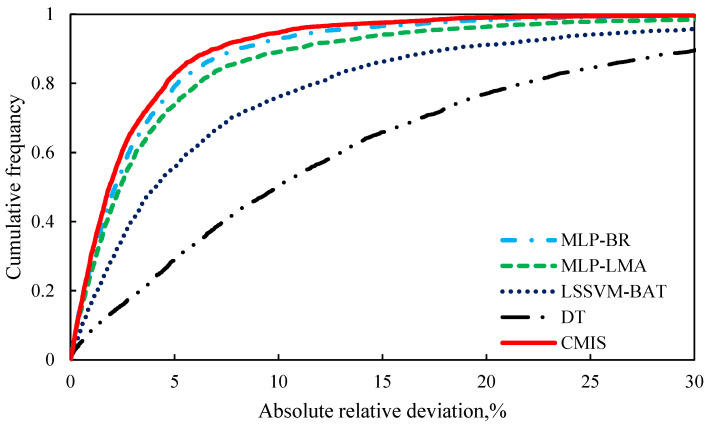
Cumulative frequency of absolute relative deviation for different models based on Model (II).

Furthermore, Figure 7 is plotted to assess the capability of the models with respect to the AARD (%) of the proposed empirical correlation (Equation (42)), smart models developed based on Model (II), Eyring’s theory in this study, and also other models form the literature. Clearly, the CMIS model and then the MLP-BR approach are more accurate, have a higher flexibility, and are more suitable to be used for the viscosity prediction of ILs approach. Figure 8 depicts the data cumulative frequency versus the ARD for the MLP-LMA, MLP-BR, LSSVM-BAT, DT, and CMIS models. As shown in this figure, the CMIS approach is more appropriate than other models and has a high accuracy in predicting viscosity of ILs. The figure implies that employing the CMIS model about 80% of the ILs viscosity data were predicted with an ARD of less than 4.5% and more than 72% of them were predicted with an ARD of less than 2.3%. 

**Figure 9:**
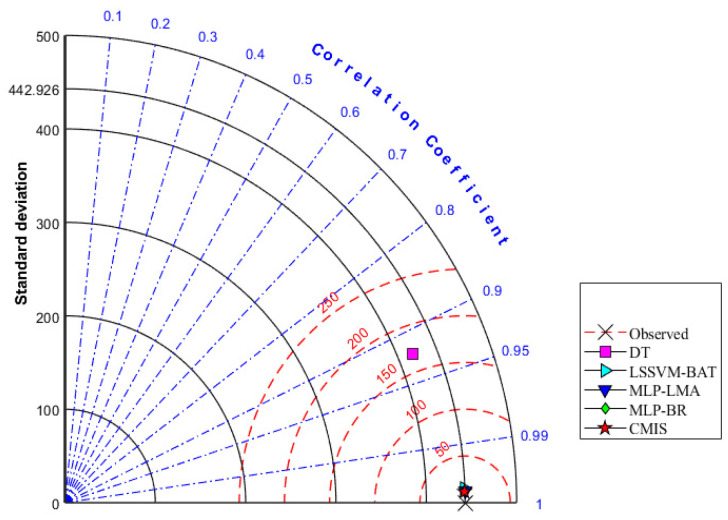
Taylor diagram for the offered methods based on Model (II).

**Figure 10:**
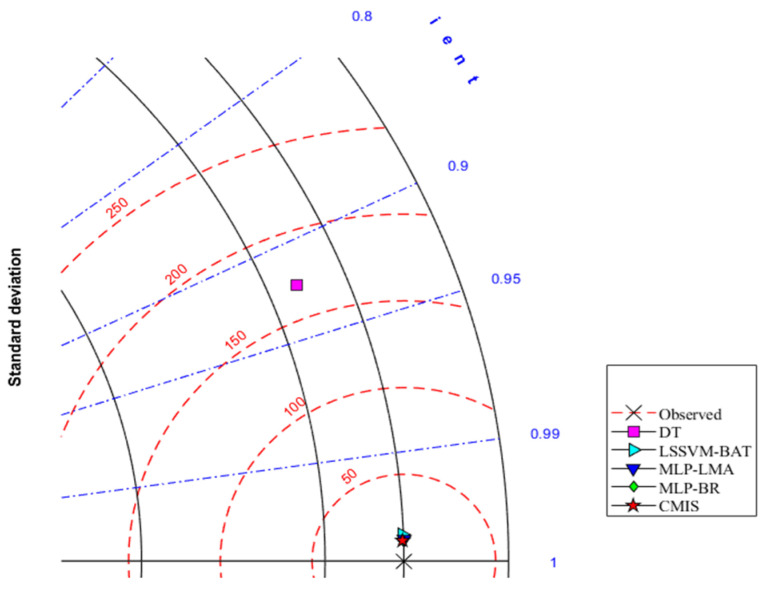
Zonation of the plotted points in the Taylor diagram based on Model (II).

Furthermore, the so-called Taylor diagram [103] is represented in Figure 9 and Figure 10 based on Model (II) to provide a more comprehensive presentation. This diagram considers various statistical parameters. As shown in Figure 9 and Figure 10, standard deviations and correlation coefficients of each model (DT, LSSVM-BAT, MLP-LMA, MLP-BR, and CMIS) are employed to quantify the variance between the predicted and measure viscosities of the ILs. RMSE is another measure considered as red circles in this diagram. An ideal predictive model is a model that has an $R^{2}$ equal to 1 in the Taylor diagram. The CMIS model exhibited the outperformance with RMSE values of 9.553 and 9.035 for the training and testing phases, respectively, while the other models showed RMSE values of more than 10. Thus, the Taylor diagram approves the outperformance of the CMIS once again as its predictions are the closest to the experimental measurements. In conclusion, the proposed model is very advantageous since it is developed based on a large dataset and the employment of various models enhances its exactness and consistency. 

**Figure 11:**
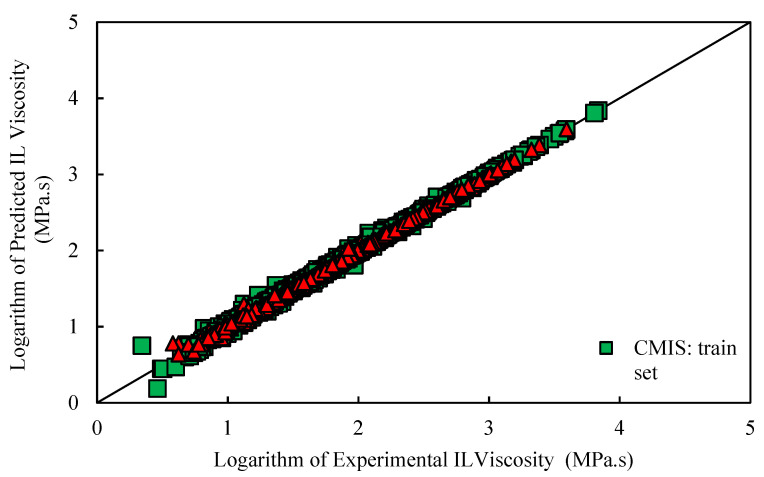
Cross plot of the proposed developed model based on Model (III) for CMIS approach.

**Figure 12:**
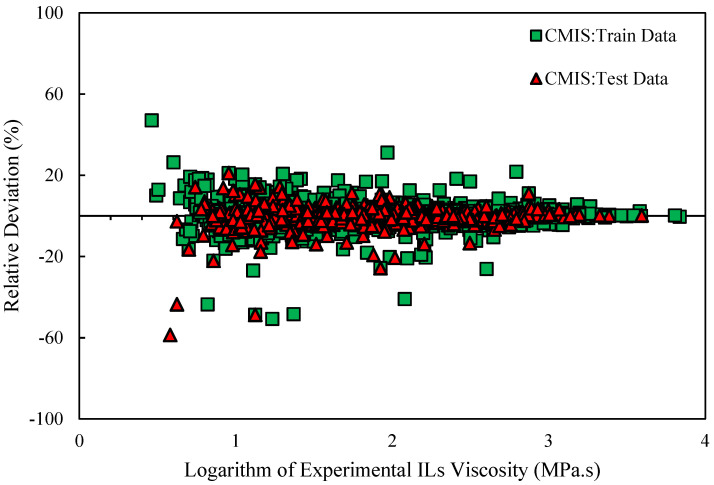
Relative deviation distribution for developed CMIS model in this study for estimation of the viscosity of ILs based on Model (III).

**Figure 13:**
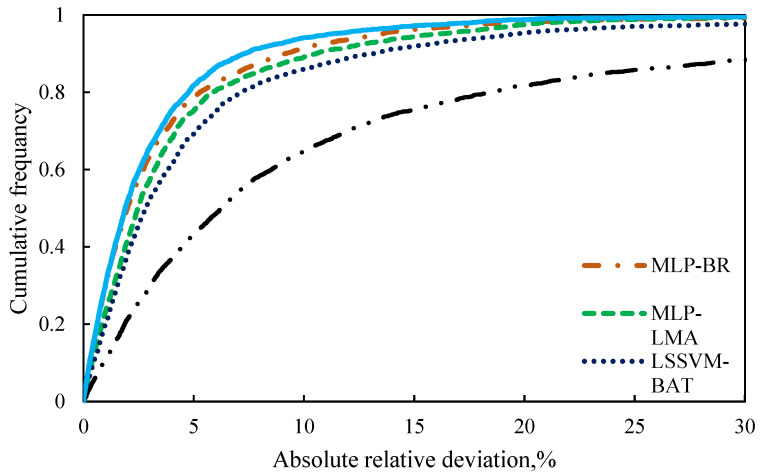
Cumulative frequency of absolute relative deviation in different models based on Model (III).

**Figure 14:**
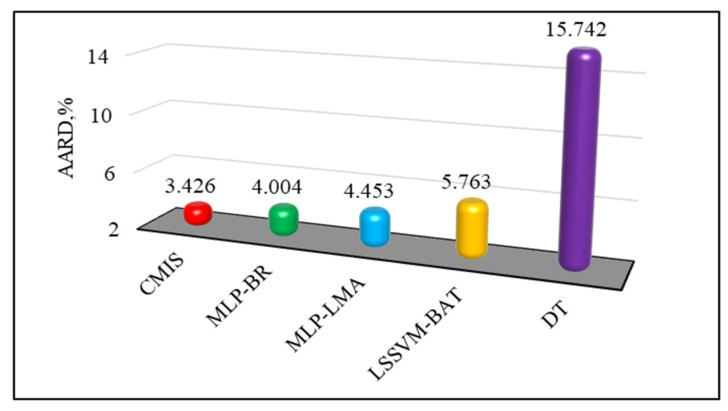
Comparison among AARD (%) of different models for prediction of viscosity of ILs based on Model (III).

On the other hand, graphical evaluation was conducted for the proposed paradigms based on Model (III) to investigate their accuracy and effectiveness. Figure 11 and Figure S6 (in the supplementary File) illustrate the logarithm of measured viscosity values versus the logarithm of estimated ones for CMIS approach and other smart models, respectively. These figures reflect that data points have formed a compact zone in the proximity of the unit slope line (45° line). In other words, the figures reveal that all intelligent models could provide consistent responses with target values. Furthermore, despite the high performance of all developed models, the constructed CMIS model could provide more reliable results thank to the satisfactory statistical criteria. Figure 12 and Figure S7 (in the Supplementary file) represent a comparison between the developed models in the regard of the relative deviations of models. Obviously, the dense concentration of points around the zero line reflects that the CMIS model is able to predict the viscosity of ILs with the lowest possible relative errors. Cumulative frequency plots are comparative plots, which are usually represented to simply clarify the competency of the models. This convenient plot has been sketched in Figure 13. As shown in this diagram, the CMIS shows the largest cumulative frequency at a specific absolute relative deviation (ARD). To clarify this, about 95% of data points were predicted by the CMIS model when we approach to the ARD of 10%, while the corresponding values for MLP-BR, MLP-LMA, LSSVM-BAT, and DT models were 88%, 83%, 80%, and 60%, respectively. Therefore, the superiority of the CMIS is once again demonstrated. In addition to the aforementioned point of Figure 13, this figure also demonstrates that the CMIS model could predict about 100% of data with lower ARD compared to the other models. Figure 14 shows the visual comparison between AARD (%) for different approaches including DT, MLP-LMA, MLP-BR, LSSVM-BAT, and CMIS for estimating the viscosity of ILs based on Model (III). As can be seen, the accuracy of CMIS-based models is generally more than other smart models.

**Figure 15:**
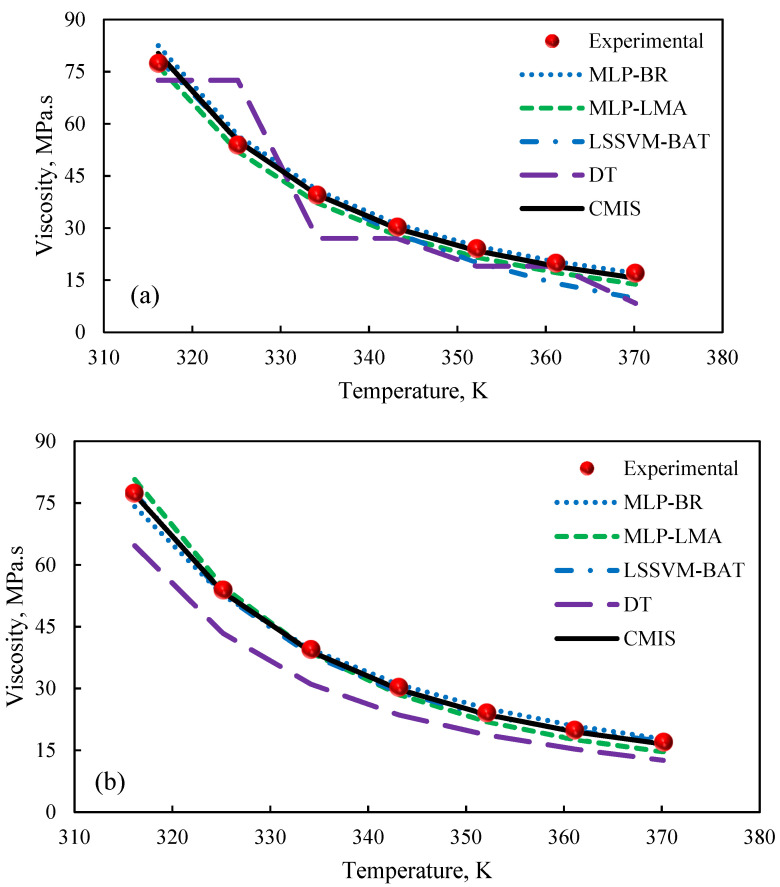
Effect of temperature on 1-ethyl-3-methylimidazolium ethylsulfate. (**a**): Model (II); (**b**): Model (III).

In order to investigate the strength and reliability of the proposed models with regard to temperature alterations, Model (II) and Model (III) were used. Figure 15a,b show the effect of temperature variations at a constant pressure (75 MPa) on 1-ethyl-3-methylimidazolium ethylsulfate based Model (II) and (III), respectively. Obviously, the predicted viscosity of ILs by all of the smart models (MLP-BR, MLP-LMA, LSSVM-BAT, and CMIS) except the DT model is in agreement to the experimental dataset. According to Figure 15, the DT approaches could not provide consistent estimations for the viscosity of ILs and the predicted values are associated with significant errors. When the temperature varies, the CMIS, MLP-BR, MLP-LMA, and LSSVM-BAT paradigms show similar physical trends. Figure 15a,b indicate a reduction in the viscosity of ILs when the temperature rises denoting an inverse relationship between the viscosity and temperature. The discussed results confirm the expected trend as well as the common knowledge of chemical thermodynamics. 

As shown in Table 3 and Table 4, among the best CMIS models for Model (II) and Model (III), the CMIS Model (II) has the lowest AARD value of 3.293%; hence, it was kept for further evaluations.

### 5.4. Identifying Outliers in Experimental Data and Applicability Domain of CMIS Model

The objective of outlier (or aberrant) detection is found for groups of data (or individual data) which deviate a lot from the bulk data in a database [104]. For this aim, the Leverage approach is one of the well-established strategies [35,104,105]. In this technique, the Hat matrix (*H*) and standardized residuals (*R*) are two main concepts [105]. The standardized residual (*R*) is calculated for each data point based on following equation:

(46) $$R_{i}=\frac{z_{i}}{(MSE{\left(1-H_{ii})\right)}^{\frac{1}{2}}}$$

where MSE denotes the model mean square error, while $z_{i}$ and $H_{ii}$ represent the error and the Hat indices (Leverage) of the *i*th data point [106]. In addition, Hat index (or Leverage) can be determined as below:

(47) $$H=X{\left(X^{t}X\right)}^{-1}\;X^{t}$$

where $X^{t}$ points out the transpose matrix, *X* represents a two-dimensional q × w matrix (where, “q” and “w” are the quantity of data points and the dimension of the model, respectively).

The Williams plot was applied in order to investigate the outliers after the measurement of the *R* and Hat index (*H*) [107,108]. In this diagram, the standardized residuals are sketched against Hats. The Leverage limit ($H^{*}$) is a parameter defined as $\frac{3a}{b}$, where b stands for the quantity of data points and a is the number of model parameters plus one [109]. Standardized residual (*R* = 3) is an acceptable data point within the range of [−3,+3] of standard deviations from the average to cover 99% of normally distributed data [35,105]. The model is statistically applicable if the large percentage of data points are within the $H^{*}\geq H\geq 0$ and 3 ≥R≥−3 [35]. Usually, data points within the range of −3 ≤ R ≤ 3 and $H^{*}$ ≤ H are regarded as “Good High Leverage” points, and are not in the applicability domain but are predicted well. By contrast, the data points with R values of higher than 3 or lower than −3 are considered as “Bad High Leverage”. These are the points predicted with large uncertainty and are located outside of the model applicability domain. These are the experimentally suspected data points. 

**Figure 16:**
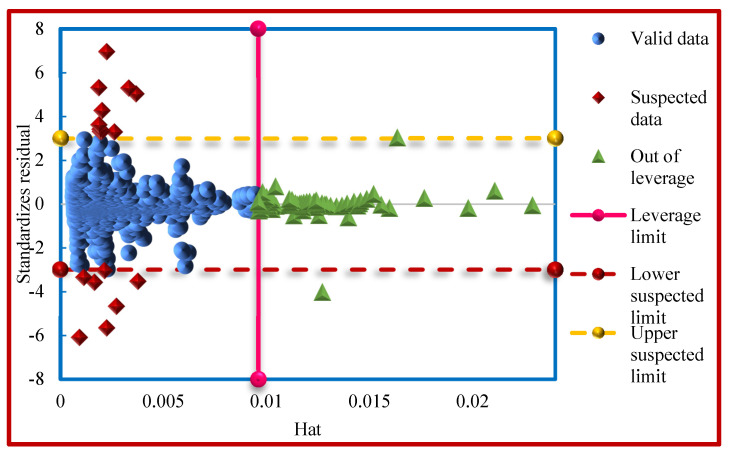
The Williams plot of the whole dataset for the CMIS model based on Model (II).

In the present study, it is clear that the performance of the CMIS as the best model based on Model (II) (thermodynamic properties, pressure, and temperature) can be significantly influenced by the consistency of the employed data. The proposed model showed a $H^{*}$ value of 0.0095. The Williams plot of the CMIS model is depicted in Figure 16. Accordingly, nearly all data points appear to be within 0 ≤ *H* ≤ 0.0095 and −3 ≤ *R* ≤ 3 reflecting that the developed CMIS system is statistically applicable. Commonly, the less standardized residual values of experimental data, the more reliable they are. Nevertheless, with respect to Figure 16, 0.63% of all data (18 suspected data points) are in the ranges of *R* < −3 or *R* > 3 and consequently, they are regarded as outliers that are associated with large uncertainty. Furthermore, 2.73% of all data (77 data points) have *H* > 0.0095. These are the points that are out of Leverage, no matter what their Hat’s (Leverage) value is, all of them are located in the range of −3 ≤ *R* ≤ 3, and consequently, they are Good High Leverage.

### 5.5. Relative Importance of Input Variables

**Figure 17:**
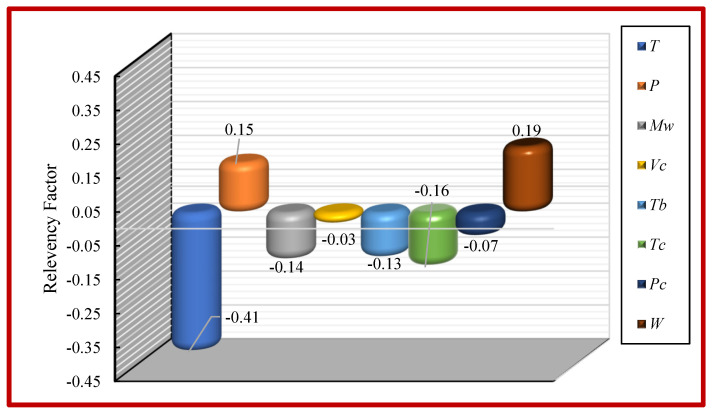
Relevancy factor of the CMIS model inputs based on Model (II).

Sensitivity analysis was performed to determine the magnitude of the impact of all input variables on the viscosity of ILs using the CMIS model. The input parameters are based on Model (II). Figure 17 shows the relative importance of the inputs. The relevancy factor (*r*) is a measure that determines the extent of the impact of each input parameter on the pure viscosity of ILs as the model output. Positive values in this technique demonstrate a direct relationship between the output and the corresponding input parameter, while negative values reveal an inverse one. The values of the relevancy factor (or *r*) are evaluated as the following equation:

(48) $$r\left(I_{i},\eta \right)=\frac{\sum_{j=1}^{n}\left(I_{ij}-{\bar{I}}_{i}\right)\left(\eta_{j}-\bar{\eta }\right)}{{\left(\sum_{j=1}^{n}{\left(I_{i,j}-{\bar{I}}_{j}\right)}^{2}\sum_{j=1}^{n}{\left(\eta_{j}-\bar{\eta }\right)}^{2}\right)}^{0.5}}$$

where n, $I_{i,j}$, and ${\bar{I}}_{i}$ are the number of datasets, the *j*th value, and mean of the i-th input variable, respectively. $\bar{\eta }$ denotes the average value of the estimated/represented viscosity of ILs, while $\eta_{j}$ expresses the *j*th value of the represented/predicted viscosity of ILs. Figure 17 depicts each parameter’s relative effect based on Model (II) on the pure viscosity of ionic liquids (as the output). As can be seen, the input parameters, namely, acentric factor and pressure, positively affect the output model. Any increase in pressure and acentric factor cause to an increase in the viscosity of pure ionic liquids. As is obvious in Figure 17, other parameters such as *T*, $M_{w}$, $V_{c}$, $T_{b}$, $T_{c}$, and $P_{c}$ show a decreasing trend due to their negative relevancy factors, which means that increasing these parameters would decrease in viscosity of ILs. Obviously, the viscosity of ILs is mainly influenced by temperature. 

## 6. Conclusions

In this study, 2813 experimental data from 45 ILs were initially modeled with the aim of obtaining a simple correlation based on temperature, pressure, molecular weight, critical volume, boiling temperature, critical temperature, critical pressure, and acentric factor of ILs. In addition, Eyring’s theory was applied based on temperature and boiling temperature to estimate the viscosity of ILs. In another scheme, the proposed models were linked to a univalent model as CMIS. To study the efficiency of the model, a comparison between the results of CMIS and experimental data was made using both statistical and graphical methods. The model showed a stable performance and high accuracy based on R2, RMSE, AARD, and ARD definitions. The effects of input parameters on model outputs were also analyzed using the relevancy factor. Finally, the leverage statistical approach was used to assess the reliability and validity of the employed dataset. In this regard, the Williams’s plot was employed to investigate the applicability domain of the constructed paradigm and find the recorded data. The results indicate that just a few data points were outside the applicability domain. It can be concluded that the developed CMIS can be used as a valuable tool to predict the viscosity of ILs with high accuracy as well as acquiring accurate knowledge of IL’s physicochemical properties in various chemical engineering processes where needed.

## Figures and Tables

**Table 1:** The selected ionic liquids in the present study.

Component of ionic liquid	Abbreviation	n	T (K)	P (MPa)
1-butyl-3-methylimidazolium hexafluorophosphate	[C_4_mim] [PF_6_]	238	273.15–413.15	0.1–249.3
1-octyl-3-methylimidazolium hexafluorophosphate	[C_8_mim] [PF_6_]	132	273.15–363.15	0.1–175.9
1-hexyl-3-methylimidazolium hexafluorophosphate	[HMIM] [PF_6_]	179	273.15–238.5	0.1–238.5
1-octyl-3-methylimidazolium tetrafluoroborate	[C_8_mim] [BF_4_]	141	273.15–363.15	0.1–224.2
1-hexyl-3-methylimidazolium tetrafluoroborate	[C_6_mim] [BF_4_]	183	283.15–368.15	0.1–121.8
1-butyl-3-methylimidazolium bis[(trifluoromethyl)sulfonyl] imide	[C_4_mim] [Tf_2_N]	344	273.15–573	0.1–298.9
1-ethyl-3-methylimidazolium bis[(trifluoromethyl)sulfonyl] imide	[C_2_mim] [Tf_2_N]	225	263.15–388.19	0.1–125.5
1-octyl-3-methylimidazolium bis[(trifluoromethyl)sulfonyl] imide	[C_8_mim] [Tf_2_N]	25	278–363.15	0.1
1-hexyl-3-methylimidazolium bis[(trifluoromethyl)sulfonyl] imide	[C_6_mim] [Tf_2_N]	236	258.15–433.15	0.1–124
1-butyl-3-methylimidazolium trifluoromethanesulfonate	[C_4_mim] [CF_3_SO_3_]	25	283.15–363.15	0.1
1-ethyl-3-methylimidazolium ethylsulfate	[C_2_mim] [EtSO_4_]	137	253.15–388.19	0.1–75
1-hexylpyridinium bis[(trifluoromethyl)sulfonyl] imide	[HPy] [Tf_2_N]	8	283–343	0–1
1-butylpyridinium bis[(trifluoromethyl)sulfonyl] imide	[BPy] [Tf_2_N]	9	283.15–353.15	0.1
1-butyl-1-methylpyrrolidinium bis[(trifluoromethyl) sulfonyl]imide	[C_4_MPyr] [Tf_2_N]	148	273.15–573	0.1–102.9
1-ethylpyridinium ethylsulfate	[EPy] [ESO_4_]	8	283–343	0.1
trimethylhexylammonium bis[(trifluoromethyl)sulfonyl]imide	[N_1116_] [Tf_2_N]	1	293.15	0.1
Trimethylbuthlammonium bis[(trifluoromethyl)sulfonyl]imide	[N_1114_] [Tf_2_N]	17	293.15–388.51	0.1
1-butyl-3-methylimidazolium tris(pentafluoroethyl) trifluorophosphate	[C_4_mim] [FAP]	1	293.15	0.1
1,2-dimethylimidasolium bis[(trifluoromethyl)sulfonyl] imide	[DMIM] [Tf_2_N]	1	298.15	0.1
trihexyl(tetradecyl)phosphonium tris(pentafluoroethyl) trifluorophosphate	[P_6,6,6,14_] [FAP]	181	268.15–373.15	0.1
1-butyl-1-methylpyrrolidinium tris(pentafluoroethyl) trifluorophosphate	[C_4_mpyrr] [FAP]	67	283.15–373.15	0.1–150
1-butyl-1-methylpyrrolidinium trifluoromethanesulfonate	[BMPyr] [TfO]	67	293.15–373.15	0.1–150
1-ethyl-3-methylimidazolium hydrogensulfate	[C_2_mim] [HSO_4_]	22	268.15–373.15	0.1
trimethylpropylammonium bis[(trifluoromethyl)sulfonyl] imide	[N_1113_] [Tf_2_N]	6	293–318	0.1
1-heptyl-3-methylimidazolium bis[(trifluoromethyl)sulfonyl] imide	[C_7_mim] [Tf_2_N]	1	293	0.1
1-undecyl-3-methylimidazolium tetrafluoroborate	[C_11_MIM] [BF_4_]	8	293–363	0.1
1-butyl-3-methylimidazolium iodid	[C_4_mim] [I]	35	289.15–388.15	0.1
1-butyl-3-methylimidazolium nitrate	[C_4_mim] [NO_3_]	27	283.15–363.15	0.1
1-dodecyl-3-methylimidazolium hexafluorophosphate	[C_12_MIM] [PF_6_]	4	333.15–363.15	0.1
1-octyl-3-methylimidazolium nitrate	[C_8_mim] [NO_3_]	16	283.15–363.15	0.1
1-hexyl-3-methylimidazolium nitrate	[C_6_mim] [NO_3_]	14	283.15–363.15	0.1
1-butylpyridinum tetrafluoroborate	[BPy] [BF_4_]	70	278.15–338.15	0.1–65.9
1-hexylpyridinium bis[(trifluoromethyl)sulfonyl] imide	[C_6_Py] [Tf_2_N]	9	298.15–398.15	0.1
1-heptyl-3-methylimidazolium hexafluorophosphate	[C_7_mim] [PF_6_]	13	293.15–263.15	0.1
1-ethyl-3-methylimidazolium diethylphosphate	[C_2_mim] [DEP]	17	292.15–373.15	0.1
1-pentyl-3-methylimidazolium hexafluorophosphate	[C_5_mim] [PF_6_]	13	293.15–263.15	0.1
1-nonyl-3-methylimidazolium hexafluorophosphate	[C_9_mim] [PF_6_]	12	303.15–363.15	0.1
1,2-dimethyl-3-propylimidazolium tetrafluoroborate	[M_1,2_P_3_im] [BF_4_]	8	289.15–343.15	0.1
1-butyl-4-methylpyridinium tetrafluoroborate	[mbpy] [BF_4_]	48	283.15–333.15	0.1–65
1,3-dimethylimidazolium dimethylphosphate	[C_1_mim] [DPO_4_]	7	293.15–323.15	0.1
1,2-dimethyl-3-propylimidazolium bis[(trifluoromethyl)sulfonyl] imide	[M_1,2_P_3_im] [Tf_2_N]	16	290–365	0.1
1-ethyl-3-methylimidazolium methylsulfate	[C_2_mim] [MSO_4_]	27	283.15–373.15	0.1
1-ethyl-3-methylimidazolium methanesulfonate	[C_2_mim] [mesy]	45	278.15–363.15	0.1
1-butyl-3-methylimidazolium perchlorate	[C_4_mim] [CLO_4_]	15	283.15–383.15	0.1
1-butyl-2,3-dimethylimidazolium tetrafluoroborate	[BDmim] [BF_4_]	7	298.15–353.15	0.1

**Table 2:** Statistical details about gathered databank in the present work.

Parameter	Symbol	Unit	Min	Max	Mean
Temperature	T	K	253.15	573	325.63
Pressure	P	MPa	0.06	298.90	24.45
Molecular Weight	M_w_	g/mole	201.22	515.13	346.65
Critical Temperature	T_c_	K	520.06	1534.63	1005.87
Critical Pressure	P_c_	bar	2.63	57.60	22.29
Critical Volume	V_c_	cm^3^/mol	550.65	2573.60	992.83
Acentric factor	Ω	-	0.21	1.10	0.59
Boiling Temperature	T_b_	K	410.77	1130.30	723.93
Experimental viscosity	η _exp_	MPa.s	1.13	9667.62	191.91

**Table 3:** Statistical parameters of the developed models for prediction of the viscosity of ILs based on Model (II).

	DT	LSSVM–BAT	MLP–LMA	MLP–BR	CMIS
Training set					
ARD%	−3.273	−0.402	−0.326	−0.170	0.011
AARD%	13.366	7.899	4.647	3.553	3.256
RMSE	152.767	11.534	8.465	8.517	9.533
SD	0.219	0.162	0.102	0.064	0.084
R^2^	0.880	0.999	0.999	0.999	0.999
Number of Data point	2250	2250	2250	2250	2250
Test set					
ARD%	−4.139	−0.190	−0.171	−1.140	0.258
AARD%	17.454	8.151	4.929	5.004	3.117
RMSE	223.793	25.356	22.368	20.044	9.035
SD	0.244	0.140	0.089	0.232	0.050
R^2^	0.751	0.994	0.997	0.997	0.999
Number of Data point	563	563	563	563	563
Total					
ARD%	−3.447	−0.345	−0.260	−0.348	−0.207
AARD%	14.184	7.941	4.707	3.841	3.293
RMSE	169.383	15.331	12.548	11.770	11.812
SD	0.225	0.158	0.099	0.118	0.083
R^2^	0.853	0.998	0.999	0.999	0.999
Number of Data point	2813	2813	2813	2813	2813

**Table 4:** Statistical parameters of the developed models for prediction of the viscosity of ILs based on Model (III).

	DT	LSSVM−BAT	MLP−LMA	MLP−BR	CMIS
Training set					
ARD%	−1.108	0.357	0.020	−0.219	−0.161
AARD%	13.589	5.552	4.522	3.768	3.422
RMSE	15.444	9.027	9.441	7.923	8.894
SD	0.292	0.129	0.114	0.076	0.073
R^2^	0.998	0.999	0.999	0.999	0.999
Number of Data point	2250	2250	2250	2250	2250
Test set					
ARD%	5.384	−0.991	−0.464	−0.977	−0.412
AARD%	24.345	6.604	4.624	4.949	3.454
RMSE	24.309	28.984	22.673	10.056	6.844
SD	1.960	0.161	0.092	0.150	0.064
R^2^	0.995	0.991	0.998	0.999	0.999
Number of Data point	563	563	563	563	563
Total					
ARD%	0.190	0.087	−0.076	−0.371	−0.172
AARD%	15.742	5.763	4.453	4.004	3.426
RMSE	17.580	15.275	13.197	8.393	9.505
SD	0.916	0.136	0.110	0.095	0.073
R^2^	0.998	0.998	0.999	0.999	0.999
Number of Data point	2813	2813	2813	2813	2813

## Data Availability

The data presented in this study are available in Supplementary Material.

## Supplementary Materials

The following are available online, Figure S1: The structure of MLPNN used in this study, Figure S2: Schematic of procedure for development of BAT-LSSVM model, Figure S3: Cross plot of the proposed correlation for viscosity of ILs, Figure S4: Logarithm of experimental viscosity data against predicted values based on Model (II), Figure S5: Relative deviation of predictions of various models versus logarithm of experimental data based on Model (II), Figure S6: Cross plot of the proposed developed models based on Artificial Neural Network based on Model (III), Figure S7: Relative deviation distribution for developed models in this study for estimation of the viscosity of ILs based on Model (III). Table S1: The adjustable parameters to proposed correlation in this study.

## Sample Availability

Samples of the compounds are not available from the authors.
