# Supplementary material for: Viscosity of Ionic Liquids: Application of the Eyring’s Theory and a Committee Machine Intelligent System
Source: Molecules. 2020 Dec 31;26(1):156. doi: 10.3390/molecules26010156 (PMC7795042; doi:10.3390/molecules26010156)
Supplement: Supplementary file 1 [file molecules-26-00156-s001.pdf]

Table S1: The adjustable parameters to proposed correlation in this study.

Symbol	Value	Symbol	Value	Symbol	Value
a	5.0711E-10	i	1.07782355	q	10.5702741
b	7.1867E+24	j	1.60057804		
c	7.3496E-10	k	1.12643237		
d	7.9665E-07	l	10.0378376		
e	7.7937E-10	m	1.00836281		
f	0.00013065	n	0		
g	304659.755	o	0		
h	3.3131E+28	p	1.12737649		

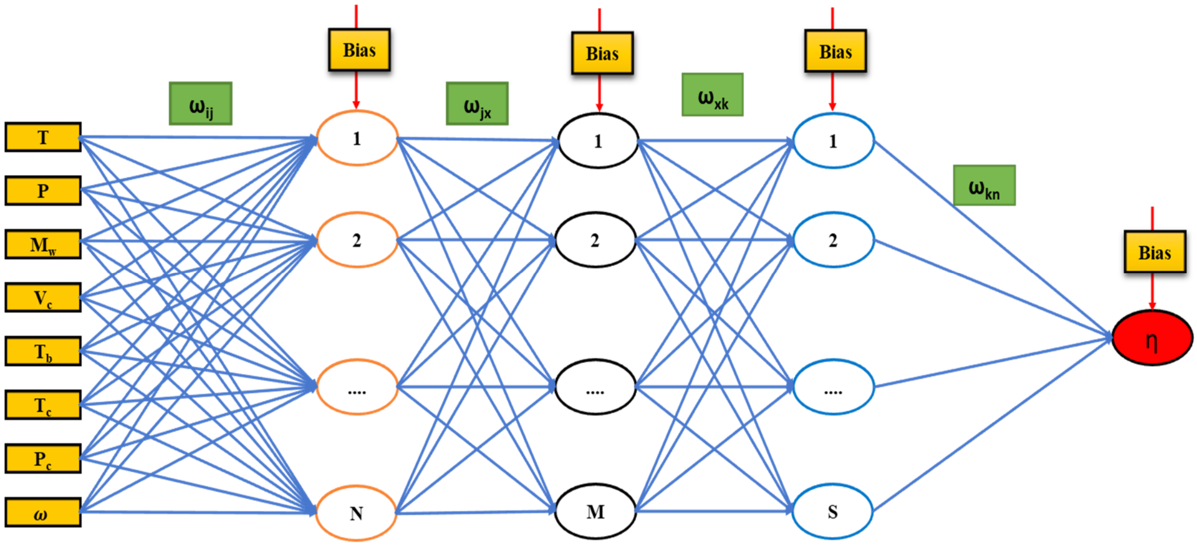

Fig. S1. The structure of MLPNN used in this study.

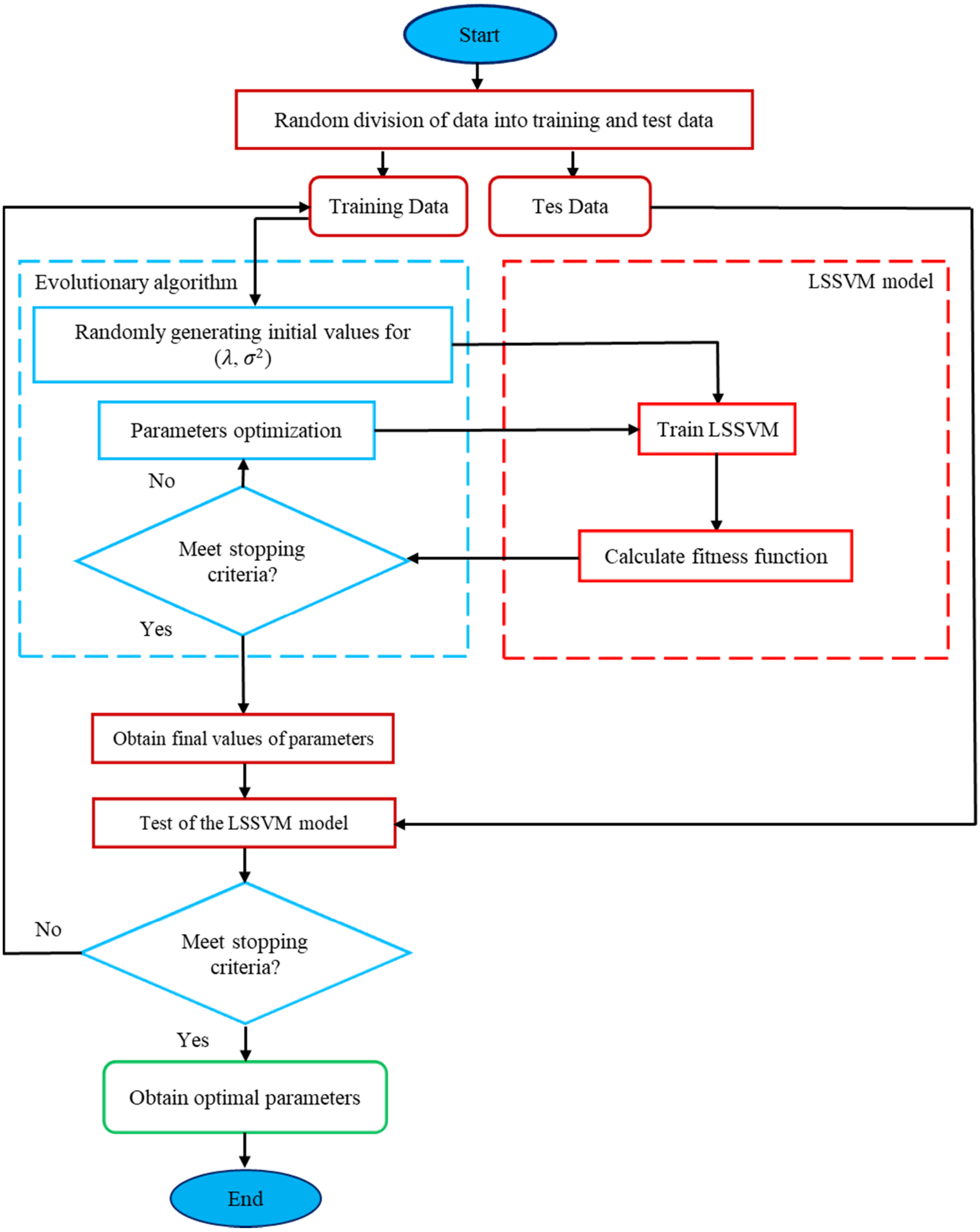

Fig.S2. Schematic of procedure for development of BAT-LSSVM model.

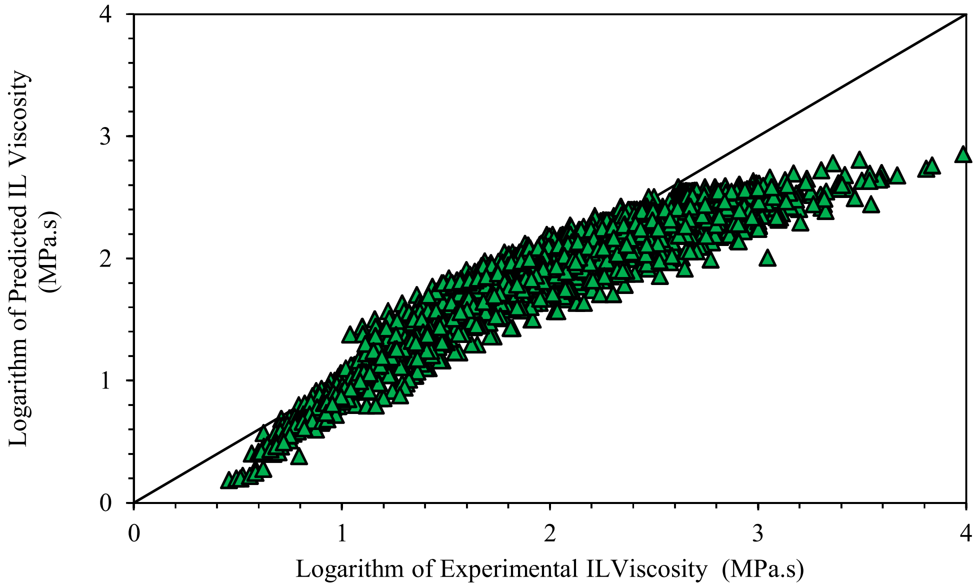

Fig.S3. Cross plot of the proposed correlation for viscosity of ILs.

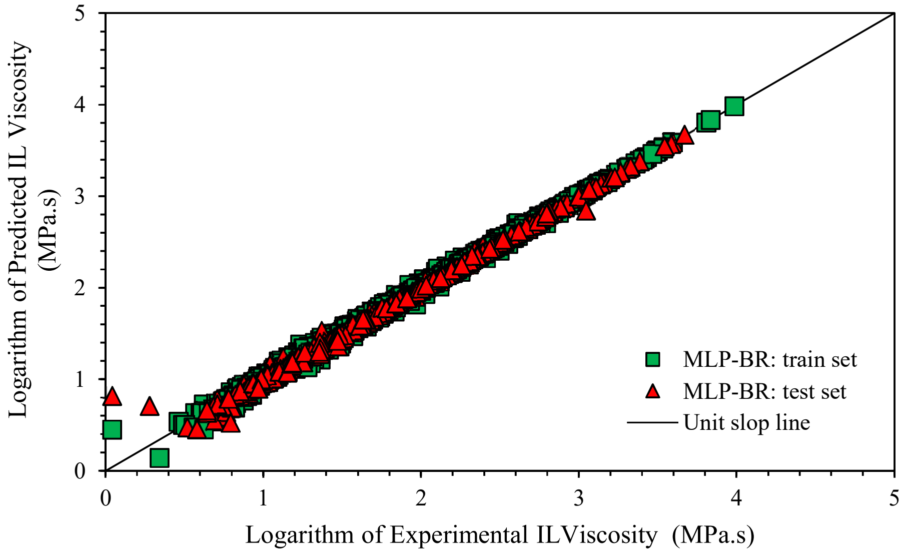

a)

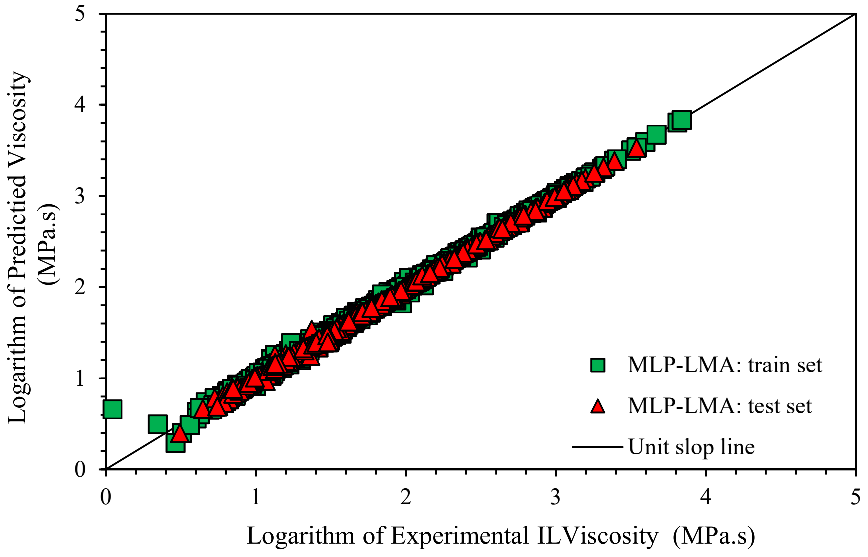
Logarithm of Predictied Viscosity (MPa.s)
Logarithm of Experimental ILViscosity (MPa.s)
MLP-LMA: train set
MLP-LMA: test set
Unit slop line
0
1
2
3
4
5

b)

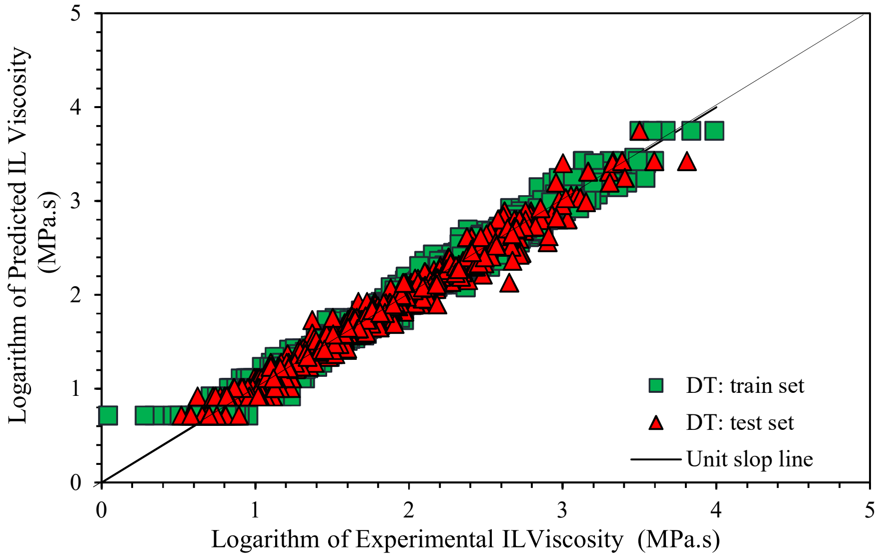
Logarithm of Predicted IL Viscosity (MPa.s)
Logarithm of Experimental ILViscosity (MPa.s)
DT: train set
DT: test set
Unit slop line
0
1
2
3
4
5

c)

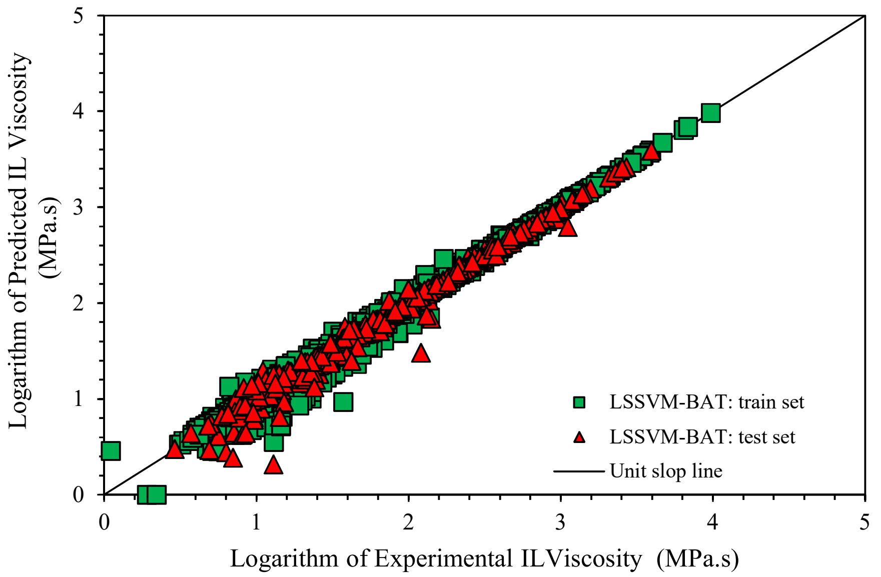

d)

Fig.S4. Logarithm of experimental viscosity data against predicted values based on Model (II): (a) MLP-BR, (b) MLP-LMA, (c) DT, (d) LSSVM-BAT.

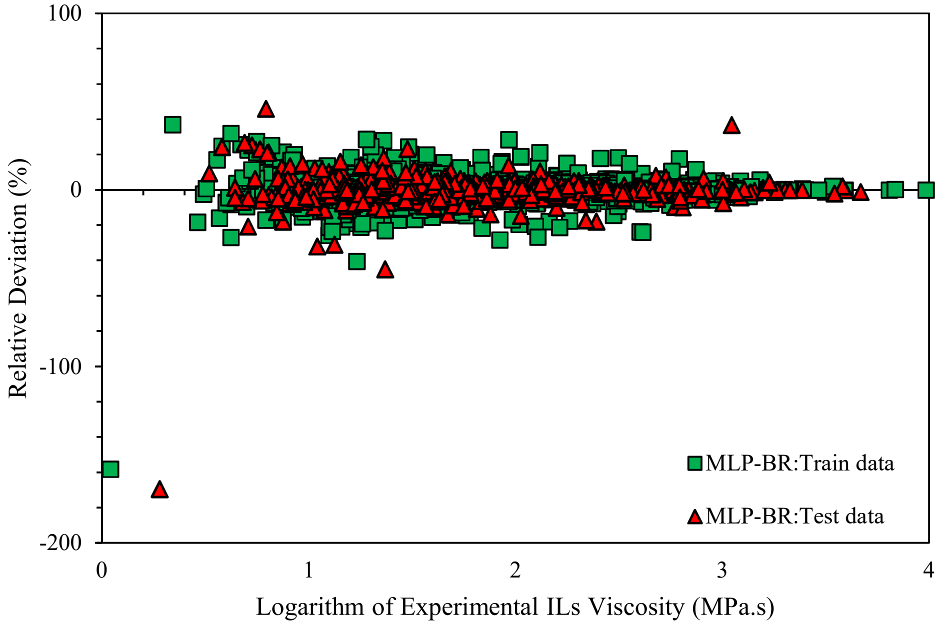

a)

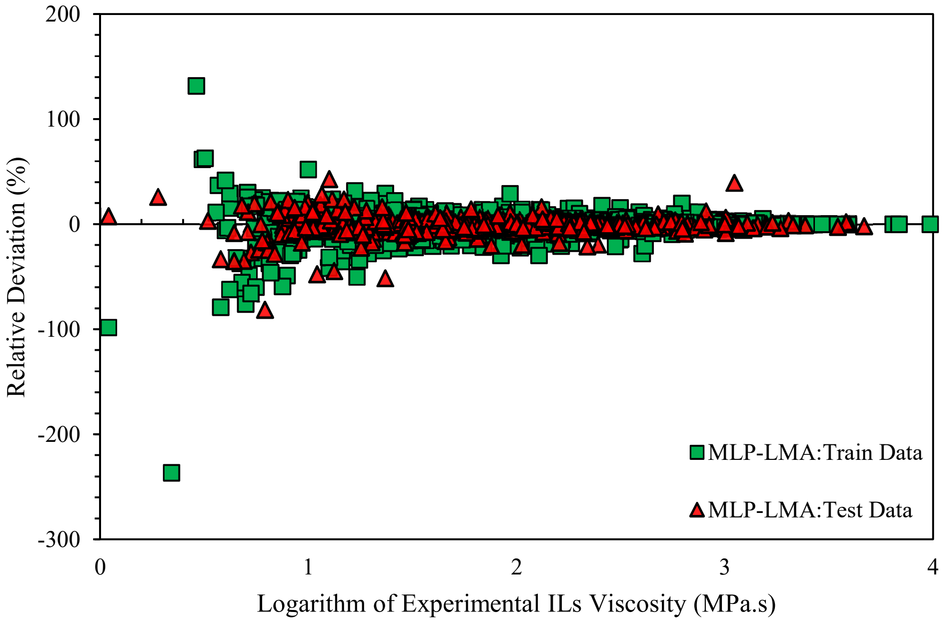
200
100
0
-100
-200
-300
Relative Deviation (%)
0
1
2
3
4
Logarithm of Experimental ILs Viscosity (MPa.s)
MLP-LMA:Train Data
MLP-LMA:Test Data

b)

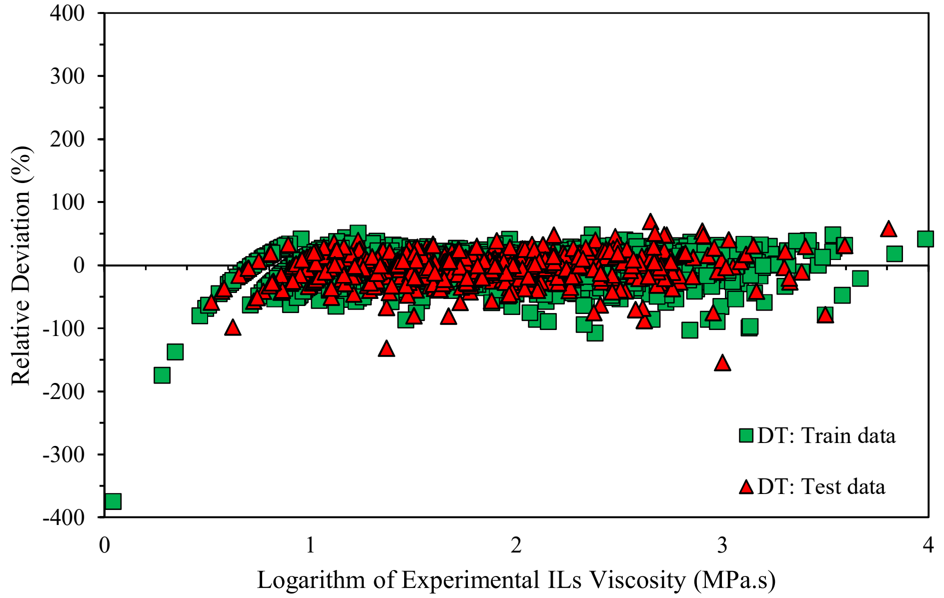
400
300
200
100
0
-100
-200
-300
-400
Relative Deviation (%)
0
1
2
3
4
Logarithm of Experimental ILs Viscosity (MPa.s)
DT: Train data
DT: Test data

c)

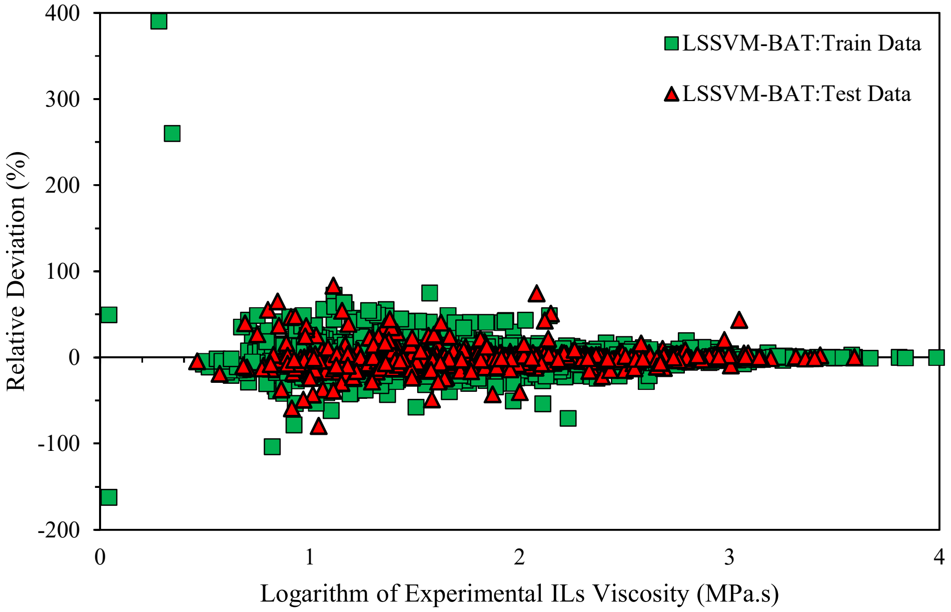

d)

Fig.S5. Relative deviation of predictions of various models versus logarithm of experimental data based on Model (II): (a) MLP-BR, (b) MLP-LMA, (c) DT, (d) LSSVM-BAT.

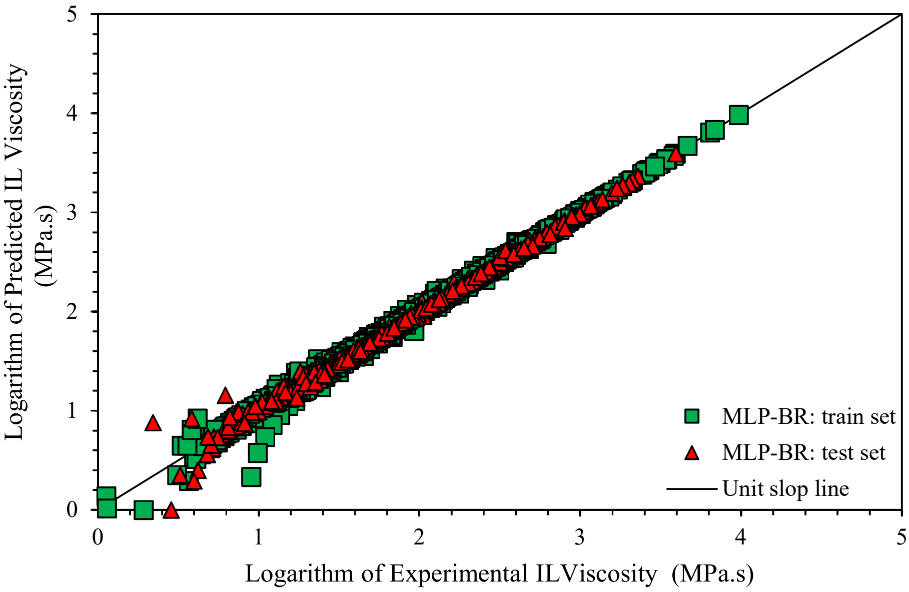

a)

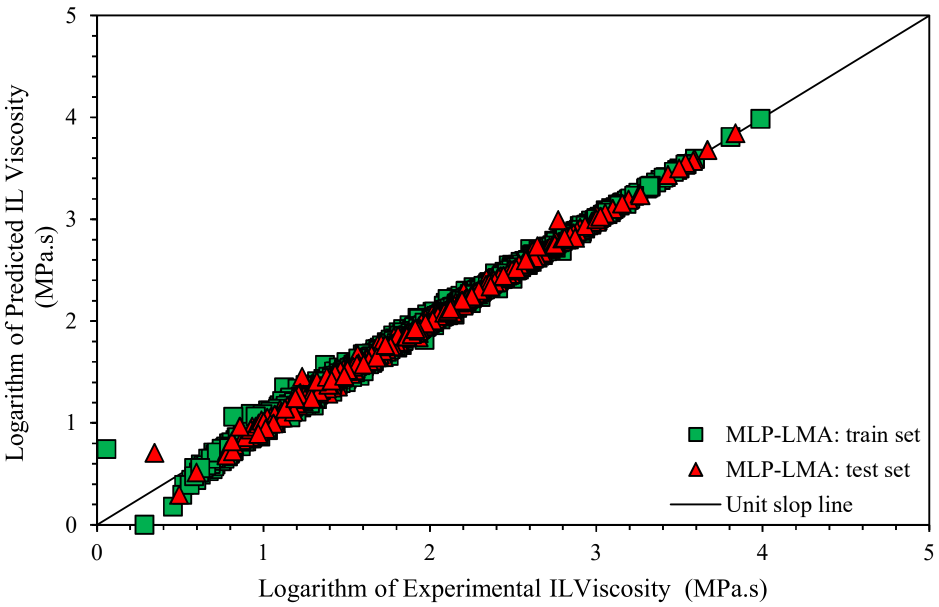

Logarithm of Predicted IL Viscosity (MPa.s)
Logarithm of Experimental ILViscosity (MPa.s)
MLP-LMA: train set
MLP-LMA: test set
Unit slop line

b)

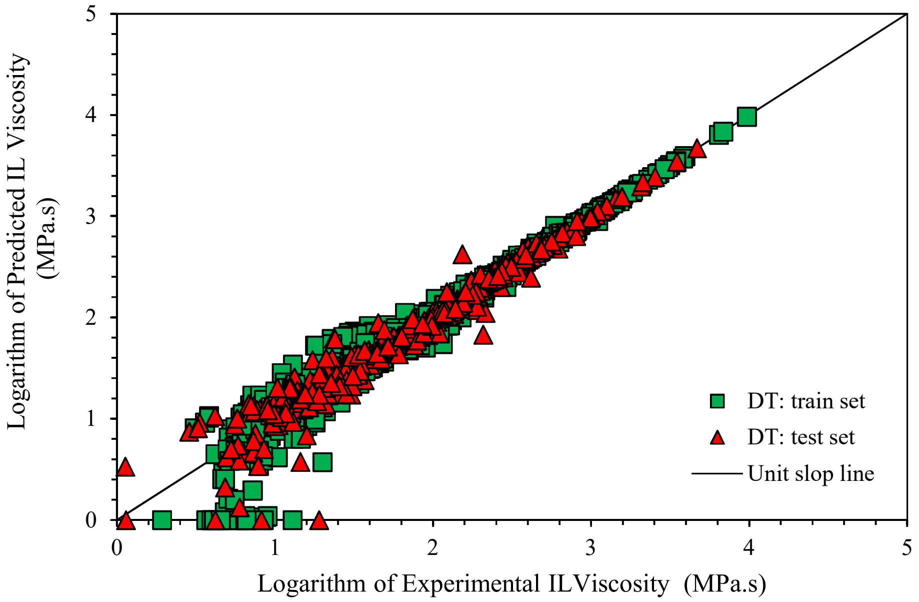

Logarithm of Predicted IL Viscosity (MPa.s)
Logarithm of Experimental ILViscosity (MPa.s)
DT: train set
DT: test set
Unit slop line

c)

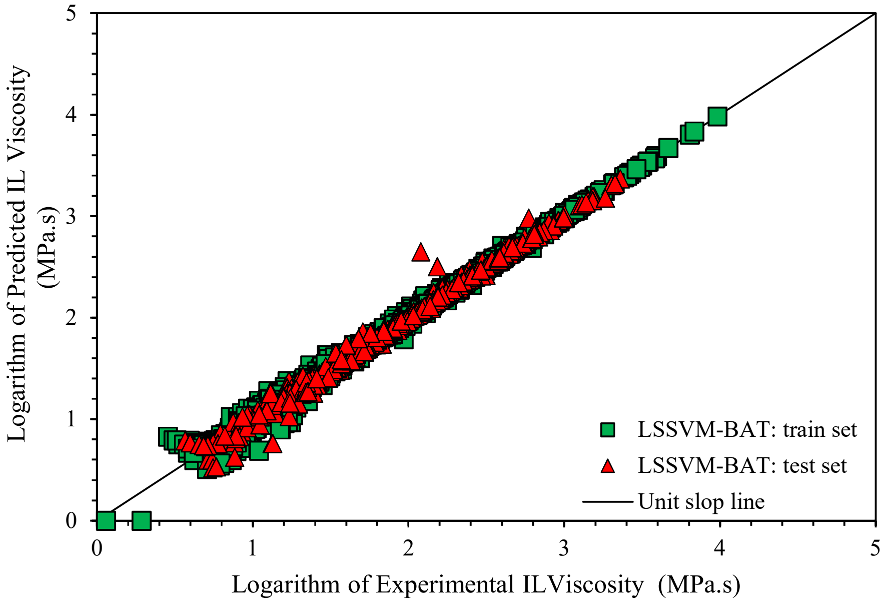

d)

Fig.S6. Cross plot of the proposed developed models based on Artificial Neural Network based on Model (III): (a) MLP-BR, (b) MLP-LMA, (c) DT, (d) LSSVM-BAT.

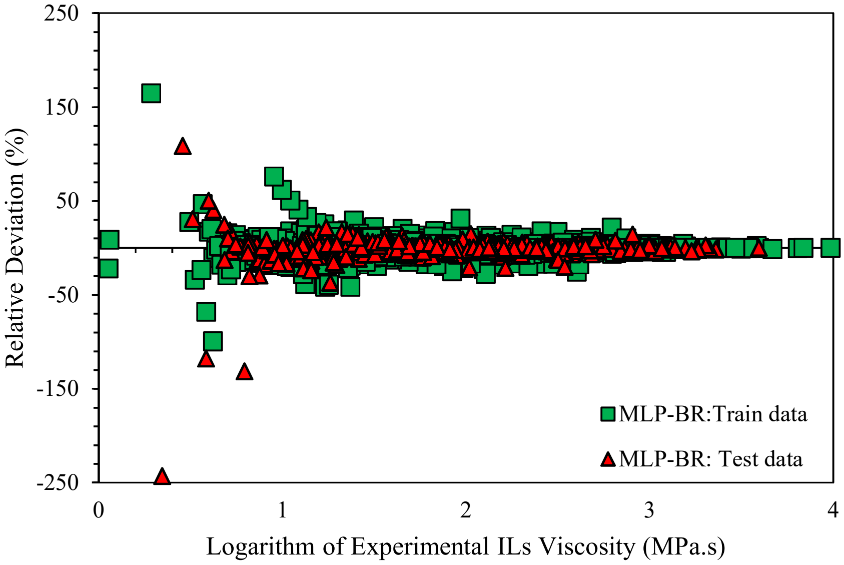

a)

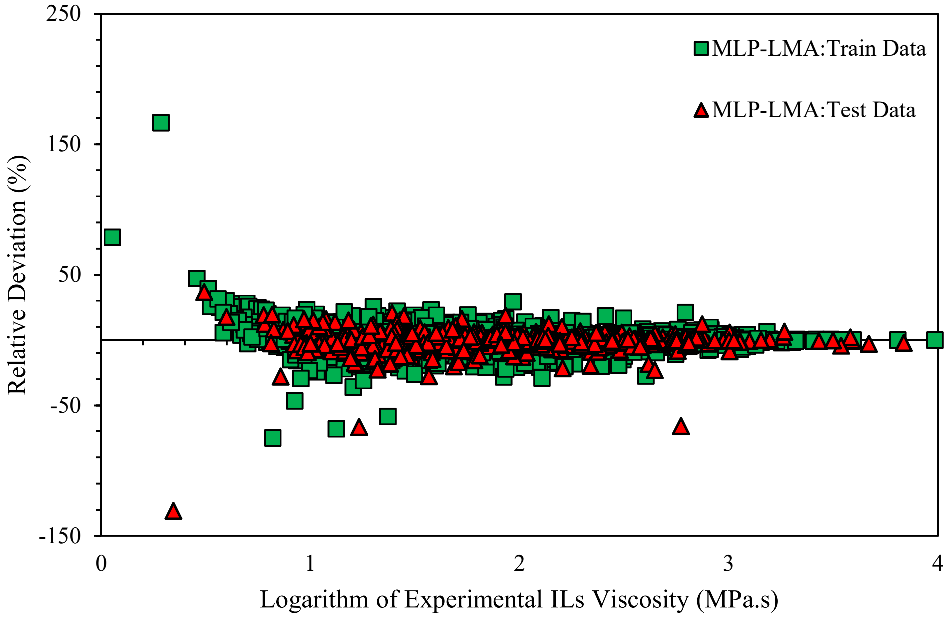
MLP-LMA:Train Data
MLP-LMA:Test Data
Relative Deviation (%)
Logarithm of Experimental ILs Viscosity (MPa.s)
250
150
50
-50
-150
0
1
2
3
4

b)

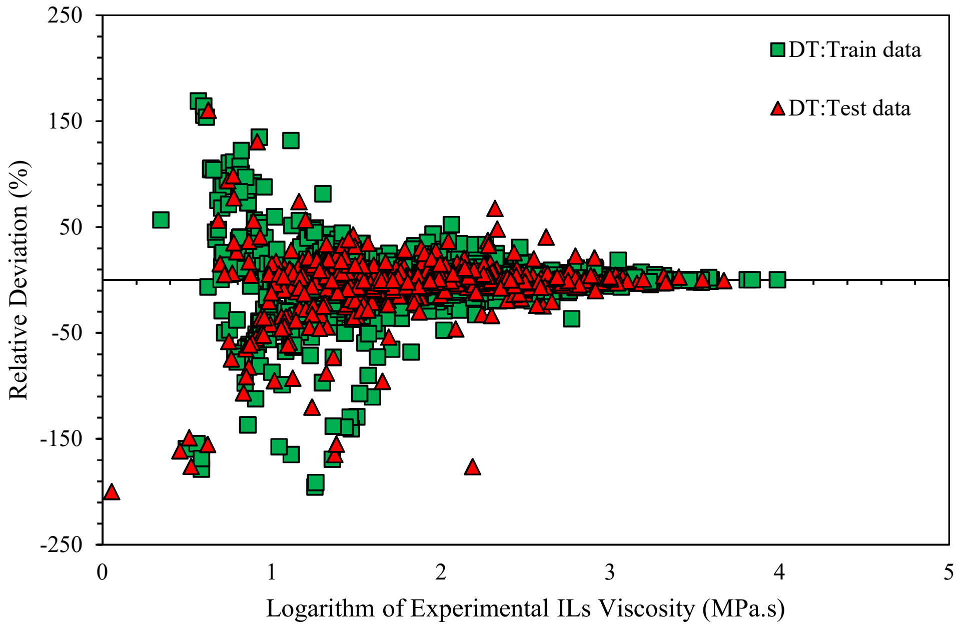
DT:Train data
DT:Test data
Relative Deviation (%)
Logarithm of Experimental ILs Viscosity (MPa.s)
250
150
50
-50
-150
-250
0
1
2
3
4
5

c)

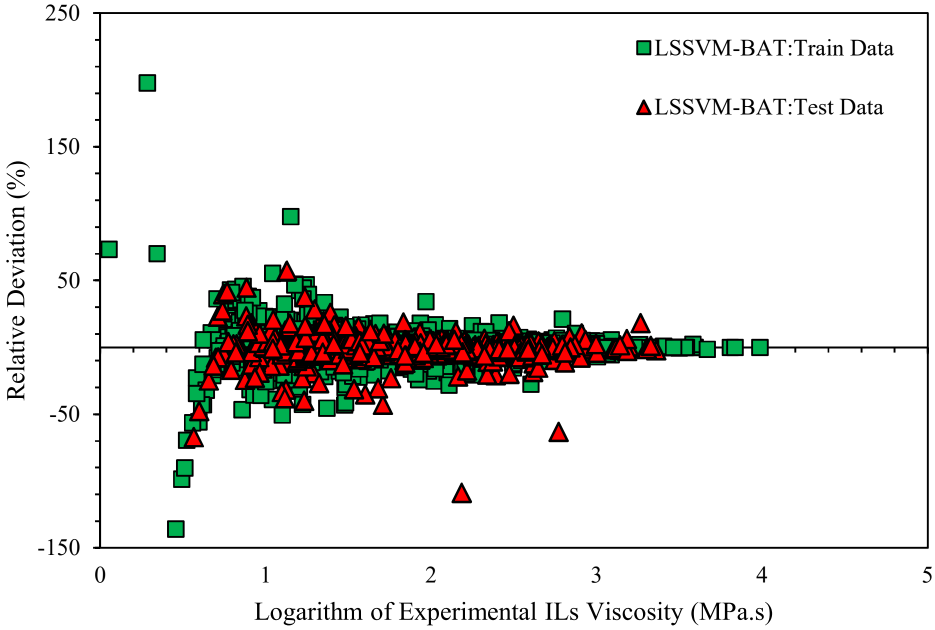

d)

Fig.S7. Relative deviation distribution for developed models in this study for estimation of the viscosity of ILs based on Model (III): (a) MLP-BR, (b) MLP-LMA, (c) DT, (d) LSSVM-BAT.
